# Reactions in Tirapazamine Induced by the Attachment of Low‐Energy Electrons: Dissociation Versus Roaming of OH

**DOI:** 10.1002/anie.202006675

**Published:** 2020-08-04

**Authors:** Eugene Arthur‐Baidoo, João Ameixa, Patrick Ziegler, Filipe Ferreira da Silva, Milan Ončák, Stephan Denifl

**Affiliations:** ^1^ Institut für Ionenphysik und Angewandte Physik Leopold-Franzens-Universität Innsbruck Technikerstrasse 25 A-6020 Innsbruck Austria; ^2^ Center for Biomolecular Sciences Innsbruck (CMBI) Leopold-Franzens-Universität Innsbruck Technikerstrasse 25 A-6020 Innsbruck Austria; ^3^ Atomic and Molecular Collisions Laboratory Department of Physics CEFITEC Universidade NOVA de Lisboa 2829-516 Caparica Portugal

**Keywords:** dissociative electron attachment, gas-phase, hypoxic cytotoxins, radiosensitizers, tirapazamine

## Abstract

Tirapazamine (TPZ) has been tested in clinical trials on radio‐chemotherapy due to its potential highly selective toxicity towards hypoxic tumor cells. It was suggested that either the hydroxyl radical or benzotriazinyl radical may form as bioactive radical after the initial reduction of TPZ in solution. In the present work, we studied low‐energy electron attachment to TPZ in the gas phase and investigated the decomposition of the formed TPZ^−^ anion by mass spectrometry. We observed the formation of the (TPZ–OH)^−^ anion accompanied by the dissociation of the hydroxyl radical as by far the most abundant reaction pathway upon attachment of a low‐energy electron. Quantum chemical calculations suggest that NH_2_ pyramidalization is the key reaction coordinate for the reaction dynamics upon electron attachment. We propose an OH roaming mechanism for other reaction channels observed, in competition with the OH dissociation.

## Graphical abstract

**Low‐energy electrons make a choice**: Upon the attachment of a low‐energy electron to the potential radiosensitizer compound tirapazamine in the gas phase, dissociation of the OH radical is by far the most abundant reaction obtained. Other dissociation channels as well as the metastable parent anion formed upon roaming of OH are computationally predicted.

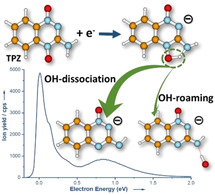

In radiation cancer treatment, high energy quanta are deposited upon the interaction of ionizing radiation with a biological medium. A large portion of this energy is channeled into the release of secondary electrons with kinetic energies of <15 eV.^1^ It has been shown that such low energy electrons (LEEs) play a key role in causing single and double strand breaks in DNA.^2^, ^3^ Sanche and co‐workers reported that LEEs induce strand breaks in the electron energy range below the thresholds of ionization and electronic excitation. The underlying mechanism of DNA damage was suggested to start with the attachment of a LEE to a DNA building block via resonant capture which leads to the formation of a transient negative ion (TNI). Subsequent bond cleavages lead to the release of a negatively charged ion and one or more neutral species. This process is known as dissociative electron attachment (DEA).^4^

Hypoxia is a characteristic feature of solid tumors representing the state of low oxygen content in cells (hypoxic cells).^5^ These cells cause limitations in the efficacy of radiation since the absence of oxygen allows restitution of radiation‐induced radical sites in DNA.^6^ Over the past decades, attempts to develop new potential drugs that can improve the sensitivity of hypoxic tumor cells towards radiation have received great attention. The production of free radicals from the chemical agent (radiosensitizer) is thereby of great importance. Free radical formation may be triggered efficiently by attachment of LEEs as shown in previous investigations with potential anticancer drugs like nimorazole (a drug of the class of nitroimidazoles) and modified pyrimidines.^7^, ^8^ The recent studies with nimorazole,^9^ comparing electron induced reactions in the gas phase and in hydrated clusters, supported the hypothesis that the mechanism of radiosensitization by this compound is based on the initial formation of the intact parent radical anion which further proceeds to cause DNA damage.

Heterocyclic aromatic N‐oxides are one class of compounds being exploited by their potential hypoxic‐selective cytotoxicity towards solid tumors.^10^ The earlier work by Brown^11^ led to the discovery of the bio‐reductive drug tirapazamine (3‐amino‐1,2,4‐benzotriazine‐1,4‐dioxide, TPZ), a derivative of the class of aromatic benzotriazene di‐N‐oxide compounds. In early stages of clinical trials, TPZ occurred to be one of the most advanced hypoxic cytotoxins and promising antitumor agent known for its selective damage to DNA in hypoxic tumor cells in vitro.^12^, ^13^ Clinical results indicated that TPZ in combination with cisplatin has increased cytotoxicity towards tumor cells in the head and neck.^14^ The antitumor potential of TPZ in hypoxic cells can be attributed to the formation of its bioactive radicals that only activate under hypoxic conditions.^15^ It was suggested that, in the biological medium, TPZ undergoes a one‐electron reduction process to form the TPZ radical anion which becomes protonated in the next reaction step (see Scheme 1). Different bioactive radicals were suggested to form subsequently from this intermediate species under hypoxic conditions. Based on an enzymatic reduction study of TPZ containing DNA, Junnotula et al.^16^ suggested the release of hydroxyl radicals from the protonated radical anion. Li et al.^17^ employed quantum chemical calculations to explore such release from the protonated radical anion in solution. Mass spectrometric experiments demonstrated the loss of OH radical from protonated tirapazamine molecules.^18^ In contrast, other studies using methods like pulsed radiolysis and electron spin resonance spectroscopy proposed the formation of the oxidizing radical benzotriazinyl (BTZ) upon loss of H_2_O from the protonated radical anion.^19^, ^20^ One of these oxidizing species—if the BTZ radical or OH radical forms as bioactive radical is still a topic of current discussions^12^—may lead to DNA double‐strand breaks by poisoning of the topoisomerase II enzyme.^6^

**Scheme 1:**
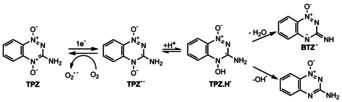
Suggested pathways for the formation of bioactive radicals from tirapazamine under hypoxic cellular conditions (see text for description). In the presence of oxygen, the TPZ radical anion is oxidized back to the precursor molecule TPZ.

So far, reactions of electrons with TPZ have been studied only in bulk solutions utilizing pulsed radiolysis.^19^ In the present study, we generated a well‐defined electron beam utilizing an electron monochromator and investigated the electron‐induced chemistry in TPZ upon attachment of a single electron to TPZ in the gas phase. We studied these reaction pathways by quadrupole mass spectrometry and quantum chemical calculations. One key fact is that a potential radiosensitizer like TPZ must be affine to electrons. In the course of the present studies, we found this property for TPZ and observed very selected efficiency of reaction pathways associated with radicals known from bulk solution.

The anion efficiency curve for electron attachment to TPZ, producing TPZ^−^, is shown in Figure 1 a. The formation of the intact parent anion TPZ^−^ occurs at the electron energy of ≈0 eV and continues up to about 0.75 eV. The observation of a parent anion under isolated conditions is usually only possible if the molecule has a positive electron affinity and can accommodate the excess internal energy released into internal degrees of freedom. In the TPZ case, the calculated vertical and adiabatic electron affinity is 1.28 and 1.57 eV, respectively (B3LYP/aug‐cc‐pVDZ).

**Figure 1:**
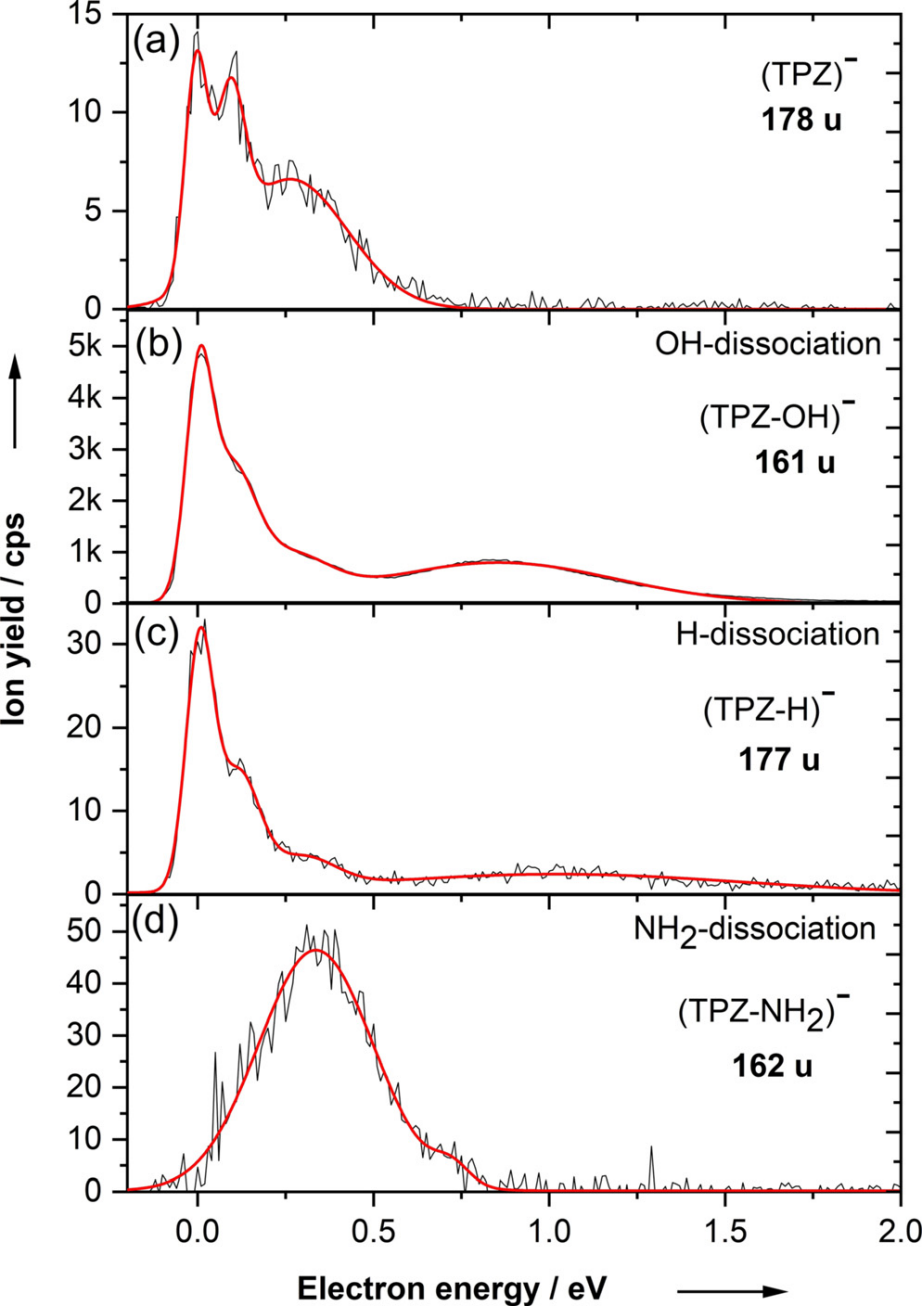
Anion efficiency curve upon electron attachment to tirapazamine (TPZ) as a function of the electron energy for the formation of (a) intact parent anion TPZ^−^, (b) OH dissociation, (c) H dissociation and (d) NH_2_ dissociation channels.

In the neutral state, the TPZ molecule is planar (*C_s_* symmetry). In the ground state and first two excited states of the anion, D_0_, D_1_ and D_2_, the NH_2_ group pyramidalizes and the molecule loses its planarity. Figure 2 shows the interpolation between the Franck–Condon point and the D_1_ minimum. It can be seen that along the NH_2_ deformation coordinate, the D_1_ state crosses with the electronic ground state of the parent molecule, and this coordinate is thus crucial for electron attachment close to 0 eV (the D_2_ minimum lies significantly closer to the Franck–Condon point and no curve crossing is observed). The crossing point in the interpolation lies at about 0.12 eV with respect to the TPZ minimum, with the calculated thermal energy of TPZ at 395 K of 0.46 eV. For D_0_ and D_1_, the attached electron is placed into an orbital delocalized over the whole molecule.

**Figure 2:**
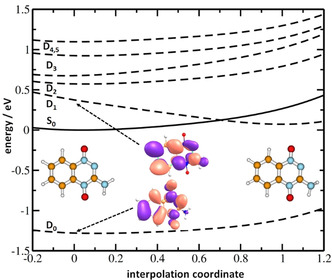
Electronic states interpolation between the planar Franck–Condon point of TPZ and D_1_ minimum of the TPZ^−^ with pyramidalized NH_2_. The ground state of TPZ (full line) and six lowest electronic states of TPZ^−^ (dashed line) are shown. Calculated at the BMK/aug‐cc‐pVDZ level of theory. For TPZ^−^, the target orbital in the ground state and the target natural transition orbital in the excited states are shown in the Franck–Condon point.

We observed in our experiments that the parent anion is not the most abundant ion formed by electron attachment to TPZ. Upon DEA, three other reaction channels with resonance positions at low electron energies are observed, namely dissociation of OH, H, and NH_2_ (the NH_2_ channel might also nominally correspond to O dissociation as discussed below), see Figure 1 b–d. The OH dissociation channel is by far the most intense one while the intensity of NH_2_ and H dissociation channels is about 100 times lower. The presence of H dissociation is well known from previous DEA investigations^21^, ^22^ with biologically relevant molecules. The peak structures are very similar for H and OH dissociation channels while the NH_2_ dissociation is shifted to higher values, showing only the peak at 0.3 eV found for the other channels as well.

In Figure 3, we show the computational analysis of the experimentally observed decomposition pathways starting from the TPZ^−^ anion. According to our calculations, all three dissociation channels share the same initial step, formation of an OH group through proton transfer from the NH_2_ group to the nearby oxygen atom, with the transition state energy well below the entrance channel (−1.23 eV at the B3LYP/aug‐cc‐pVDZ level of theory). Following the transfer reaction, the OH group might dissociate directly, with the overall reaction energy of −0.61 eV. The exothermicity and the low barrier of the proton transfer reaction explain the high intensity of the OH dissociation channel observed in the experiment.

**Figure 3:**
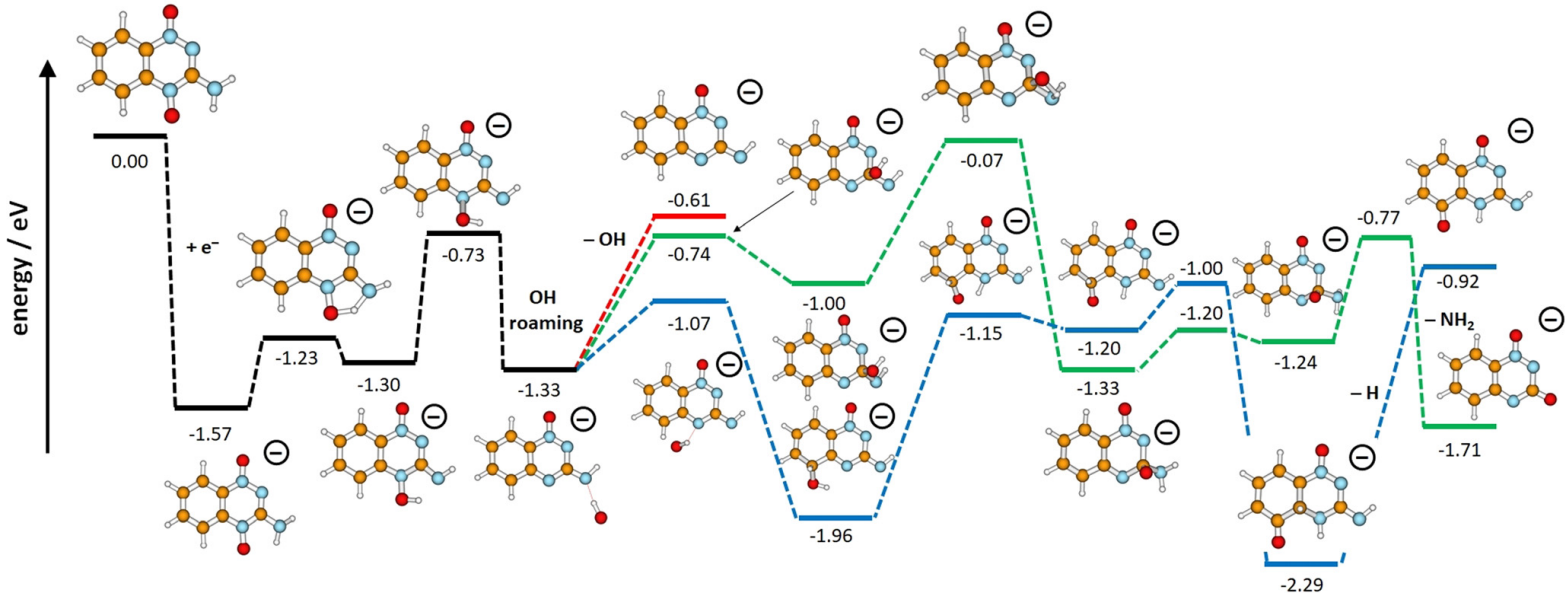
Suggested pathways for OH, H and NH_2_ dissociation channels (red, blue and green pathway, respectively). Calculated at the B3LYP/aug‐cc‐pVDZ level of theory. See the Supporting Information for comparison to M06‐2X results.

The minor channels leading to H and NH_2_ dissociation are more complicated. The calculations show that the H dissociation channel cannot proceed through direct dissociation of any hydrogen atom from the parent molecule as the respective thermodynamic thresholds are at least 0.92 eV (see the Supporting Information), being incompatible with the ion yield observed at ≈0 eV, as shown in Figure 1 c. To obtain an exothermic reaction (−0.92 eV), an H atom has to dissociate from a C−H bond and be replaced by an oxygen atom. Similarly, direct NH_2_ dissociation is an endothermic reaction with a threshold of 2.00 eV and only the structure in which an oxygen atom has moved to the site of the dissociated NH_2_ group leads to an exothermic reaction of −1.71 eV.

Therefore, we suggest that the minor dissociation channels appear due to OH roaming mechanism. The roaming of small entities like H, CH_3_ or NO_2_ was previously proposed for photochemical reactions or as a result of bimolecular collisions.^23^ Roaming is characterized by an elongated bond distance of the entity where the covalent bond is already broken and the complex is held by van der Waals forces.^24^ As shown in Figure 3, when the OH group forms (black path) it dissociates most of the time (red path). However, in a minority of cases, it might stay in the vicinity of the molecule and attach to another atom. When it attaches to a carbon atom (blue path), it forms even a more stable complex and, after another proton transfer reaction, it dissociates the hydrogen atom, with oxygen staying on the benzene ring. The overall reaction is by 0.3 eV more exothermic compared to OH dissociation. For NH_2_ dissociation, the initial step is the attachment of the OH group to the carbon atom bearing the NH group (green path), eventually leading to regeneration of the NH_2_ group and its dissociation. The NH_2_ dissociation channel is the most exothermic one, however with a high‐lying transition state.

Finally, since the experiment does not resolve isobaric ions with very similar mass, the NH_2_ dissociation channel could nominally correspond also to O dissociation. However, our calculations show that ^1^O dissociation does not take place directly (with dissociation energy of 3.16 eV) and stays endotherm even after certain molecular rearrangement (2.01 eV, see Figure S2). If ^3^O would dissociate instead of ^1^O, violating thus spin conservation, the dissociation energy would drop to 0.04 eV for the most stable isomer found.

The suggested reaction pathways can account for all experimental observations. As mentioned above, the OH dissociation channel is the least exothermic one but is observed with the highest intensity. The lower abundance of the other reaction channels may be explained by the competition between the dissociation and spontaneous emission of the excess electron (autodetachment). The latter will be more probable with increased complexity of reactions in the TNI. In addition, to dissociate NH_2_ we have to surpass a high barrier. This barrier may lead to the shift of the ion yield for NH_2_ dissociation to slightly higher electron energies. If the OH group does not dissociate directly, it may form a metastable TPZ^−^ anion with the OH group attached to another carbon atom. By its excess energy it is also prone to autodetachment and thus only a fraction of TPZ^−^ anions reach the detector.

If we compare the present results for TPZ in the gas phase with the proposed (more complex) solution phase chemistry upon reduction, we note that at isolated conditions a single LEE is able to predominantly form the hydroxyl radical which was also suggested to form as a final bioactive radical of TPZ in solution.^16^ The other bioactive radical suggested—the benzotriazinyl radical—may also form upon DEA which yields OH^−^ as negatively charged product in the dissociation process. However, the presently observed ion yield of OH^−^ is very low (about 0.1 % relative to (TPZ‐OH)^−^, see Figure S1 in the Supporting Information). This clear preference of excess charge localization at the BTZ radical may result from the significantly higher electron affinity of this species (2.97 eV) compared to the OH radical (1.83 eV^25^). Our calculations show that the situation will change with protonation or hydration (see Figure S3 in the Supporting Information): When H^+^ attaches to TPZ^−^, the BTZ radical may be produced in an exothermic reaction with the energy of −0.70 eV. Microhydration of TPZ^−^ also supports OH^−^ + BTZ formation compared to OH radical evaporation. While for non‐hydrated TPZ^−^, OH evaporation is favored by 1.1 eV compared to OH^−^ dissociation, the difference drops to 0.2 eV for the TPZ^−^.(H_2_O)_2_ complex. Finally, the roaming mechanism should be also affected considerably by the presence of a solvent, with possible suppression of minor reaction channels as the OH radical might be more constrained in its roaming in the vicinity of the molecule due to the presence of solvent molecules.

In conclusion, we have studied electron attachment to TPZ in the gas phase using mass spectrometry as well as quantum chemical calculations and found very selective unimolecular dissociation upon attachment of a single electron to TPZ. The dominating reaction channel of TPZ reduced by LEEs leads to the emission of an OH radical, while the abundance of the complementary reaction channel with emission of BTZ radicals is about three orders of magnitude lower. In this case, the unimolecular chemistry in TPZ may be driven by the corresponding electron affinities of the involved moieties. The other channels observed could result from the proposed OH roaming mechanism. The possible use of TPZ as radiosensitizer is emphasized by its electron‐affine nature observed in the present study.

## Conflict of interest

The authors declare no conflict of interest.

## Supporting information

As a service to our authors and readers, this journal provides supporting information supplied by the authors. Such materials are peer reviewed and may be re‐organized for online delivery, but are not copy‐edited or typeset. Technical support issues arising from supporting information (other than missing files) should be addressed to the authors.

Supplementary
