# Supplementary material for: Reactions in Tirapazamine Induced by the Attachment of Low‐Energy Electrons: Dissociation Versus Roaming of OH
Source: Angew Chem Int Ed Engl. 2020 Aug 4;59(39):17177–81. doi: 10.1002/anie.202006675 (PMC7540495; doi:10.1002/anie.202006675)
Supplement: Supplementary file 1 — Supplementary [file ANIE-59-17177-s001.pdf]

Angewandte Chemie
Eine Zeitschrift der Gesellschaft Deutscher Chemiker

Supporting Information

Reactions in Tirapazamine Induced by the Attachment of Low-Energy Electrons: Dissociation Versus Roaming of OH

Eugene Arthur-Baidoo, João Ameixa, Patrick Ziegler, Filipe Ferreira da Silva, Milan Ončák, and Stephan Denifl**

anie_202006675_sm_miscellaneous_information.pdf

Table of Contents

Section S1: Experimental and theoretical methods …………………………………………………………………………… 1

Section S2: Negative ion mass spectrum…………………………………………………………………………… 2

Section S3: Additional computational data …………………………………………………………………………… 3

Section S4: Cartesian Coordinates (in Å) and Energies (in Hartree) of Molecules and Ions Calculated at the B3LYP/aug-cc-pVDZ Level……………….. 6

References …………………………………………………………………………… 19

Author Contributions …………………………………………………………………………... 20

Section S1

Experimental and theoretical methods

The present study has been carried out using a crossed electron-molecule beam apparatus, described in detail in ref[1]. In brief, the setup consisted of a hemispherical electron monochromator (HEM) acting as the electron beam source, which was coupled with a quadrupole mass analyser. The tirapazamine sample ($C_7H_6N_4O_2$, 178 u) with a stated purity of ≥ 98% (HPLC) was purchased from Sigma Aldrich (Austria) and was used as delivered. The effusive molecular beam of TPZ produced from the resistively heated oven connected to a capillary was crossed perpendicularly with the electron beam produced by the HEM. The compound was sublimed at the oven temperature of ~395 K. The incident electron current amounted to 8–19 nA. The used electron energy resolution of ~100 meV (full-width-half-maximum) ensured the best compromise between beam intensity and energy resolution. For calibration of the electron energy scale, the well-known 0 eV peak in the ion yield of Cl^- formed upon electron attachment to CCl_4 was used. The step width was 10 meV in the shown electron energy scans.

Quantum chemical calculations in the electronic ground states were performed using density functional theory (DFT) calculations using B3LYP and M06-2X[2] functionals along with the aug-cc-pVDZ basis set. Wavefunction stabilization was performed for each calculation. For excited states, time-dependent DFT (TDDFT) with the BMK functional and Coupled Cluster Singles and Doubles Equation of Motion (EOM-CCSD) methods were used. Due to overestimation within the DFT theory, the singlet-triplet gap of the oxygen atom (1.97 eV) was taken from the NIST Atomic Spectra Database.[3] See the SI for benchmarks. All calculations were performed in the Gaussian software.[4]

Section S2

Negative ion mass spectrum

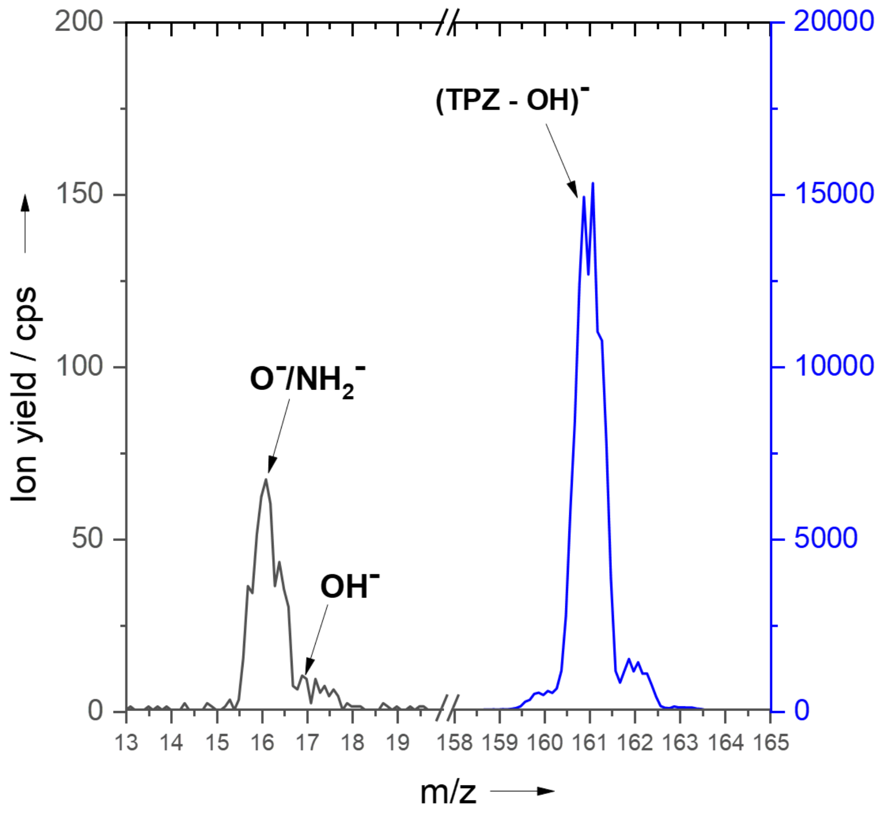

Figure S1. Negative ion mass spectrum of tirapazamine in the m/z ranges between 13 and 20 and between 158 and 165, respectively. The spectrum was obtained by the sum of individual mass spectra recorded in the electron energy range of 0 to 8 eV in steps of 1 eV. The spectrum reflects the large intensity difference between the complementary OH^- and $(TPZ\text{-}OH)^-$ anions formed by electron attachment to tirapazamine.

Section S3

Additional Computational Data

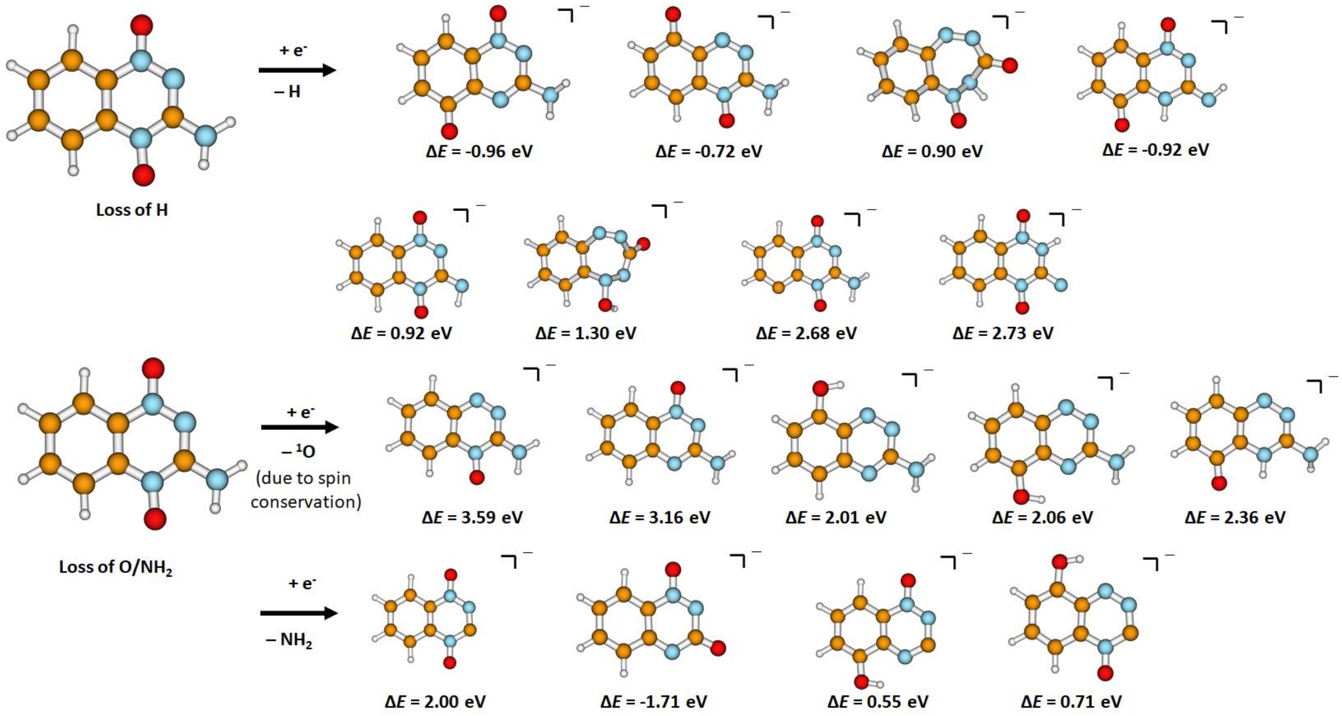

Figure S2. Dissociation energies for H, O and NH_2 dissociation channels for various target isomers. Calculated at the B3LYP/aug-cc-pVDZ level of theory.

Table S1. Energies (in eV) along the path in Figure 3 calculated using two different DFT functionals along with the aug-cc-pVDZ basis set.

point \ functional	B3LYP	M06-2X
black, 1	0.00	0.00
black, 2	-1.57	-1.63
black, 3	-1.23	-1.34
black, 4	-1.30	-1.45
black, 5	-0.73	-0.52
black, 6	-1.33	-1.24
red, 1	-0.61	-0.52
green, 1	-0.74	-0.51
green, 2	-1.00	-1.04
green, 3	-0.07	-0.03
green, 4	-1.33	-1.23
green, 5	-1.20	-1.12
green, 6	-1.24	-1.17
green, 7	-0.77	-0.67
green, 8	-1.71	-1.55
blue, 1	-1.07	-0.85
blue, 2	-1.96	-2.05
blue, 3	-1.15	-1.12
blue, 4	-1.20	-1.14
blue, 5	-1.00	-0.99
blue, 6	-2.29	-2.23
blue, 7	-0.92	-1.01

Table S2 compares TDDFT excited state energies to the ones provided by the higher-level EOM-CCSD method. Among the tested DFT functionals, the best results are provided by the BMK functional, with the average absolute error of 0.20 eV for first six excited states.

Table S2. Excited state energies (in eV) of TPZ^{-} in the TPZ optimal structure (B3LYP/aug-cc-pVDZ) calculated using various methods employing the aug-cc-pVDZ basis set.

State	EOM-CCSD	TD-BMK	TD-CAM-B3LYP	TD-BHandHLYP
1A’	1.79	1.65	1.72	1.95
1A’’	1.60	1.85	1.98	2.06
2A’’	1.74	1.95	2.08	2.16
3A’’	2.03	2.20	2.42	2.50
2A’	2.51	2.38	2.39	2.58
4A’’	2.28	2.58	2.68	2.79

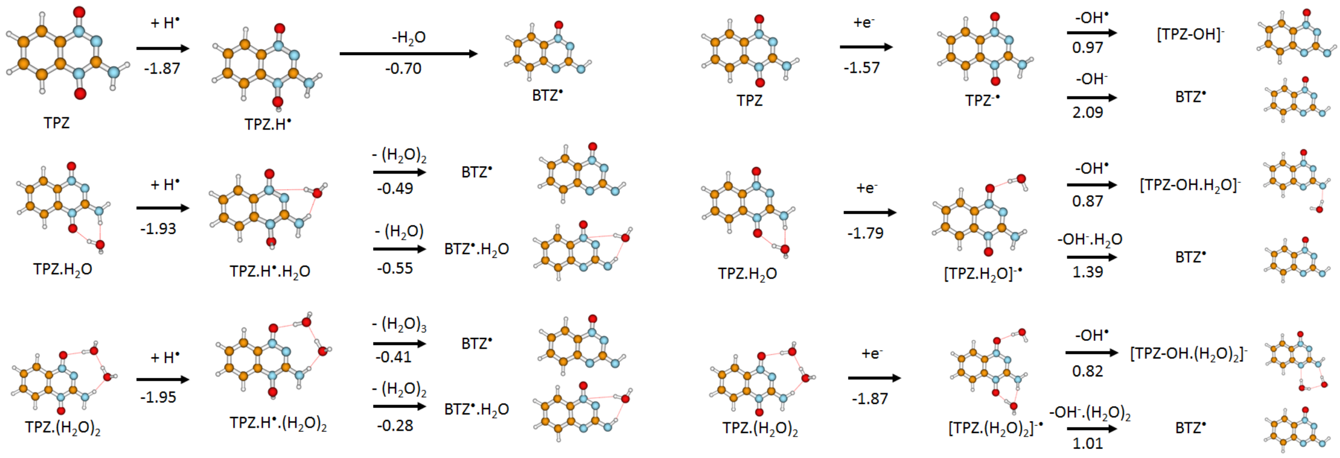

Figure S3. Reaction energies (in eV) of hydrogen atom addition and electron attachment to TPZ, and of BTZ and $[TPZ\text{-}OH]^{-}$ formation, including microhydrated clusters. Calculated at the B3LYP/aug-cc-pVDZ level of theory.

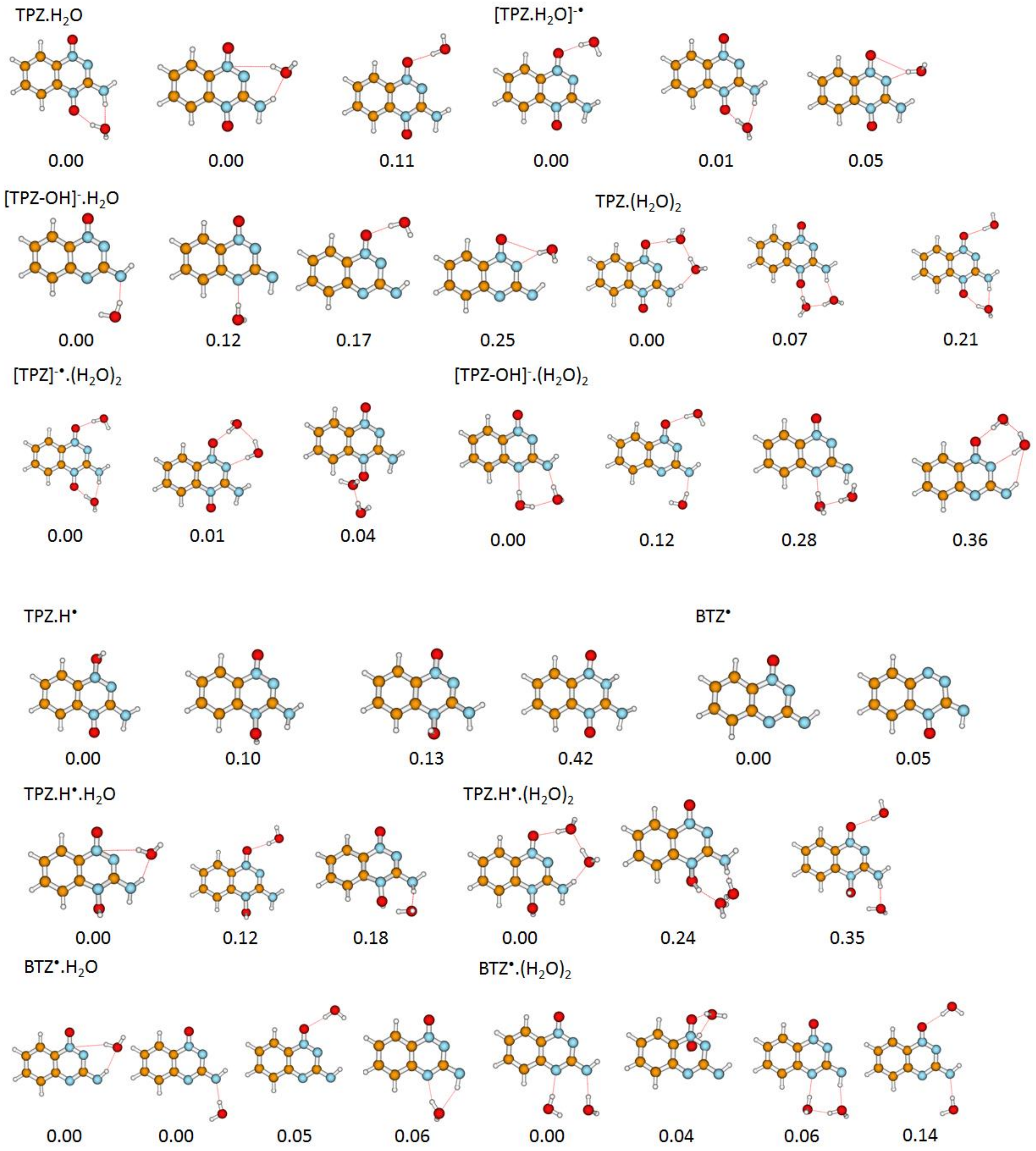

Figure S4. Relative energy of various isomers (in eV). The respective isomers with the minimum energies were considered for the calculation of the reaction energies shown in Fig. S3, with the exception of TPZ.H where the second lowest isomer is considered due to suggested mechanism (dissociation of a water molecule). Calculated at the B3LYP/aug-cc-pVDZ level of theory.

Section S4

Cartesian Coordinates (in Å) and Energies (in Hartree) of Molecules and Ions Calculated at the B3LYP/aug-cc-pVDZ Level

```
H
E = -0.501657
H 0.000000 0.000000 0.000000

[M-H]-
E = -639.175466
N 0.713870 -1.333377 0.001503
C -0.382624 -0.521104 0.000598
C -0.212906 0.863939 0.000256
N 1.135716 1.334935 0.000082
N 2.174660 0.543566 -0.000071
C 2.001432 -0.848280 -0.000158
C -1.313605 1.748209 -0.000138
C -2.577547 1.164856 -0.000351
C -2.776209 -0.230267 -0.000310
C -1.696071 -1.159564 0.000036
O -1.766184 -2.429654 -0.000194
O 1.328488 2.574186 0.000102
N 3.011482 -1.662818 -0.000898
H -3.788660 -0.638290 -0.000600
H -3.454532 1.817131 -0.000599
H -1.151767 2.820757 -0.000364
H 3.865964 -1.105329 -0.001073
H 0.525647 -2.333398 -0.000526

[M-H]-
E = -639.177197
C -1.708357 -1.205507 0.006329
C -0.355649 -0.577490 -0.006610
C -0.230357 0.846729 -0.008514
C -1.335336 1.717841 -0.009778
C -2.599132 1.123550 -0.008853
C -2.790764 -0.261551 -0.001438
N 1.076902 1.361950 0.004019
N 2.141934 0.574792 -0.001841
C 1.903629 -0.757785 -0.017593
N 0.736531 -1.373041 -0.008428
O 1.263831 2.609347 0.026004
N 3.060868 -1.544103 -0.086841
H -3.800264 -0.677260 0.001814
H -3.479364 1.773523 -0.011854
H -1.181915 2.791474 -0.009427
H 2.897557 -2.487409 0.243160
H 3.877102 -1.101001 0.316275
O -1.855202 -2.453252 0.022797

[M-H]-
E = -639.168240
C 0.743567 1.862121 -0.003044
C 0.016415 0.676080 -0.007895
C 0.640239 -0.625923 -0.002498
C 2.136276 -0.731058 -0.001145
C 2.822765 0.542569 0.002868
C 2.150148 1.753770 0.002009
N -0.072473 -1.757860 0.014768
N -1.397948 -1.708637 0.006041
C -2.011201 -0.512319 -0.017977
N -1.372164 0.680843 -0.006776
N -3.383320 -0.455301 -0.077118
H 2.736919 2.677724 0.004728
H 3.913366 0.507998 0.006340
O 2.731192 -1.828141 -0.001521
H -3.739550 0.458940 0.185517
H -3.848502 -1.276206 0.284308
H 0.222649 2.814879 -0.003026
O -2.067791 1.792130 0.017749

[M-H]-
E = -639.107928
N -1.047519 -1.018186 0.000293
C 0.269184 -0.717204 0.000109
C 0.691849 0.642205 -0.000019
N -0.299388 1.640192 0.000154
N -1.568337 1.356067 0.000100
C -2.008712 0.033576 -0.000189
C 2.055454 0.977775 -0.000202
C 3.013664 -0.024350 -0.000282
C 2.603349 -1.384155 -0.000117
C 1.268719 -1.734400 0.000074
O 0.061297 2.859742 0.000334
N -3.275849 -0.213027 -0.000707
O -1.479142 -2.260492 0.000428
H 0.929594 -2.767068 0.000176
H 3.360058 -2.171783 -0.000208
H 4.073705 0.230159 -0.000432
H 2.315404 2.033587 -0.000260
H -3.359407 -1.234892 -0.000487

[M-H]-
E = -639.041637
N 0.958317 -1.215811 -0.190013
C -0.323130 -0.725907 -0.106258
C -0.582836 0.690112 -0.025320
N 0.442748 1.575632 -0.149581
N 1.667122 1.037918 -0.491498
C 2.085946 -0.256940 0.153144
C -1.911526 1.160264 0.152240
C -2.960774 0.268030 0.200622
C -2.719022 -1.131557 0.080779
C -1.434466 -1.612695 -0.062144
O 0.338157 2.846637 -0.018135
N 3.130838 -0.459683 0.742684
O 1.176925 -2.474892 -0.228542
H -1.218280 -2.676587 -0.130026
H -3.557169 -1.829793 0.114885
H -3.980463 0.635962 0.323580
H -2.057537 2.235588 0.226692
H 2.374470 1.746628 -0.301245

[M-H]-
E = -639.043289
N -1.034792 -1.019814 -0.000950
C 0.363353 -0.795396 -0.004144
C 0.761638 0.580475 -0.006764
N -0.185097 1.613964 0.002992
N -1.475336 1.344952 -0.014068
C -1.857092 0.060336 -0.026047
C 2.132365 0.915873 -0.007084
C 3.046132 -0.121538 -0.004000
C 2.604019 -1.472614 0.000614
C 1.265336 -1.892950 -0.000040
O 0.170860 2.832519 0.029798
N -3.206378 -0.214606 -0.087522
O -1.587665 -2.191346 0.033932
H 3.396429 -2.235683 0.001333
H 4.117268 0.109851 -0.006713
H 2.415133 1.964494 -0.008757
H -3.380155 -1.184072 0.170817
H -3.797530 0.499438 0.315107

[M-H]-
E = -639.108608
C -2.951918 0.710536 -0.338680
C -1.714020 1.292703 -0.385050
```

C -0.550727 0.533722 -0.016710
C -0.654224 -0.913469 0.161975
C -1.994832 -1.431952 0.290933
C -3.104063 -0.664433 0.053277
N 0.589361 1.185561 0.248964
N 1.635201 0.451468 0.860027
C 2.231264 -0.502876 -0.129995
N 1.542904 -1.639440 -0.354455
N 0.299703 -1.848883 0.061221
O 0.788857 2.435354 0.056137
H -1.575540 2.342469 -0.635115
H -3.835995 1.302621 -0.583534
H -4.101690 -1.102030 0.117705
H -2.075630 -2.496985 0.511511
O 3.361088 -0.235151 -0.547708
H 2.350223 1.165977 0.997208

[M-H]-
E = -639.094194
C 2.874557 -0.980384 -0.385150
C 2.897036 0.415954 -0.484379
C 1.757896 1.171184 -0.185851
C 0.570474 0.531056 0.191090
C 0.549155 -0.880255 0.317153
C 1.705715 -1.616971 0.041866
N -0.638337 1.184177 0.539855
O -0.488785 2.604008 0.475165
N -0.541645 -1.572344 0.940982
N -1.738137 -1.253848 0.751375
C -2.128328 -0.345138 -0.372371
O -2.934890 -0.882024 -1.153037
N -1.656576 0.889886 -0.445075
H 1.769900 2.256663 -0.247530
H 3.811769 0.925851 -0.794077
H 3.767045 -1.565564 -0.611908
H 1.670776 -2.697460 0.187554
H -0.966254 2.796867 -0.355173

[M-NH2]-
E = -583.697775
N -0.986991 1.420411 -0.000017
C 0.198346 0.708702 0.000029
C 0.156196 -0.706815 -0.000027
N -1.081542 -1.323545 -0.000030
N -2.204899 -0.591993 0.000222
C -2.225069 0.774674 0.000227
C 1.353317 -1.460439 -0.000053
C 2.569471 -0.807115 0.000005
C 2.619062 0.613306 0.000084
C 1.458137 1.359741 0.000091
O -1.160440 -2.598352 -0.000256
O -0.922754 2.715279 -0.000183
H 1.452691 2.447198 0.000126
H 3.586951 1.118317 0.000136
H 3.496365 -1.382935 -0.000004
H 1.266811 -2.544430 -0.000110

[M-NH2]-
E = -583.834197
C -1.490038 -1.665199 -0.000009
C -0.279366 -0.893838 -0.000033
C -0.448041 0.520944 -0.000018
C -1.708154 1.140777 0.000020
C -2.852961 0.358926 0.000034
C -2.727500 -1.055588 0.000018
N 0.739762 1.292043 -0.000025
N 1.912199 0.741829 -0.000041
C 2.023629 -0.693190 -0.000144
N 0.914785 -1.499377 -0.000060
O 0.637742 2.555195 0.000073
H -1.387223 -2.750625 -0.000020
H -3.629377 -1.672726 0.000030
H -3.838945 0.824671 0.000061
H -1.747482 2.227878 0.000036
O 3.178557 -1.136153 0.000121

[M-NH2]-
E = -583.751259
N 0.103604 -1.832480 -0.000067
C -0.354614 -0.561663 0.000022
C 0.467888 0.576952 -0.000038
N 1.843629 0.312019 -0.000197
N 2.304545 -0.930745 -0.000260
C 1.449811 -2.060204 -0.000206
C -0.070072 1.885263 0.000053
C -1.446830 2.024342 0.000195
C -2.315703 0.897280 0.000253
C -1.777441 -0.373529 0.000172
O -2.521257 -1.508168 0.000235
O 2.649693 1.289903 -0.000215
H -3.398183 1.029241 0.000368
H -1.885197 3.023895 0.000264
H 0.604340 2.737247 0.000003
H -1.829136 -2.216472 0.000181

[M-NH2]-
E = -583.745296
C 0.055627 1.892167 -0.000043
C -0.496784 0.585203 -0.000026
C 0.348567 -0.546230 0.000024
C 1.771855 -0.363715 0.000065
C 2.307809 0.905687 0.000054
C 1.430337 2.028938 -0.000004
N -0.110581 -1.809183 0.000046
N -1.433659 -1.996776 -0.000003
C -2.368031 -0.990199 -0.000055
N -1.857661 0.325834 -0.000053
O -2.661147 1.325482 -0.000121
O 2.536198 -1.488612 0.000108
H -0.618967 2.743857 -0.000082
H 1.867945 3.029410 -0.000015
H 3.389478 1.040155 0.000085
H 1.878163 -2.218610 0.000076

[M-OH]-
E = -563.925271
N -0.331663 1.723639 0.000000
C -0.816463 0.482818 0.000000
C 0.000000 -0.691451 0.000000
N 1.401189 -0.475965 0.000000
N 1.906157 0.713617 0.000000
C 1.036801 1.850916 0.000000
C -0.525849 -1.991536 0.000000
C -1.901645 -2.174312 0.000000
C -2.749257 -1.033985 0.000000
C -2.235563 0.245787 0.000000
O 2.169743 -1.483905 0.000000
N 1.603365 3.027621 0.000000
H -2.884393 1.121950 0.000000
H -3.833216 -1.174450 0.000000
H -2.325982 -3.178607 0.000000
H 0.169387 -2.828303 0.000000
H 2.614776 2.878852 0.000000

[M-OH]-
E = -563.816159
N -0.304018 1.715129 0.000000
C -0.819724 0.468025 0.000000
C 0.000000 -0.693781 0.000000
N 1.413297 -0.496944 0.000000
N 1.923406 0.703742 0.000000
C 1.047360 1.790684 0.000000
C -0.534852 -1.983958 0.000000
C -1.918642 -2.127569 0.000000
C -2.763306 -0.992499 0.000000
C -2.231703 0.282916 0.000000
O 2.158613 -1.493500 0.000000
N 1.590987 3.008248 0.000000
H -2.858580 1.173193 0.000000
H -3.844730 -1.129339 0.000000
H -2.355330 -3.125950 0.000000
H 0.137410 -2.838219 0.000000

H 2.611833 2.894189 0.000000

[M-O]-
E = -564.506872
N 1.008923 0.742586 -0.009373
C -0.349394 0.445686 -0.008854
C -0.697236 -0.947506 0.000724
N 0.220156 -1.946731 0.016586
N 1.544351 -1.592043 0.000688
C 1.882192 -0.312519 -0.023176
C -2.088774 -1.260139 0.004044
C -3.054607 -0.254697 0.001737
C -2.682201 1.100564 -0.002646
C -1.321125 1.450880 -0.006995
N 3.216047 0.054950 -0.086994
O 1.460190 1.991505 0.029499
H -0.985335 2.485707 -0.008299
H -3.445124 1.881018 -0.002694
H -4.113273 -0.525834 0.004848
H -2.365855 -2.314931 0.011627
H 3.332503 1.025478 0.196969
H 3.836086 -0.628435 0.326198

[M-O]-
E = -564.522547
C 1.475541 -1.694722 0.001214
C 0.324136 -0.884370 -0.004779
C 0.512725 0.534942 -0.004107
C 1.804858 1.096612 0.000698
C 2.926470 0.256561 0.005088
C 2.763743 -1.134401 0.006047
N -0.618945 1.338750 -0.003554
N -1.887965 0.792902 0.001056
C -1.927149 -0.540374 -0.016031
N -0.935404 -1.436832 -0.008791
N -3.227088 -1.073169 -0.077134
H 1.331502 -2.776153 0.000166
H 3.638590 -1.788406 0.009680
H 3.927703 0.692633 0.008186
H 1.891337 2.180543 0.001477
H -3.269624 -1.999492 0.330750
H -3.922237 -0.433781 0.289448
O -0.524176 2.620952 0.006309

[M-O]-
E = -564.564981
N 1.448738 1.153229 -0.008598
C 0.089960 0.910312 -0.004327
C -0.363802 -0.440799 -0.003303
N 0.477820 -1.511755 0.002519
N 1.825608 -1.248160 0.003531
C 2.196035 0.041261 -0.016463
C -1.759067 -0.715736 0.000204
C -2.692579 0.315267 0.004089
C -2.234774 1.649282 0.005056
C -0.866039 1.947114 0.001323
H -0.513819 2.979959 0.000320
N 3.587899 0.245669 -0.079493
H -2.964308 2.462066 0.008032
H -3.758014 0.081233 0.006882
O -2.131220 -2.038174 0.002018
H 3.857630 1.129145 0.337247
H 4.101152 -0.548505 0.285190
H -1.271750 -2.511584 0.001002

[M-O]-
E = -564.563201
N 1.023851 0.964809 -0.010150
C -0.189151 0.304260 -0.003656
C -0.259654 -1.119127 -0.002366
N 0.875179 -1.880583 0.001361
N 2.076819 -1.209246 0.004114
C 2.070515 0.127434 -0.015962
C -1.546540 -1.728394 0.001744
C -2.696789 -0.942887 0.006009
C -2.627386 0.466030 0.006230
C -1.369627 1.072405 0.001059
O -1.233751 2.443898 -0.001434
N 3.343190 0.727644 -0.078920
H -3.527613 1.080944 0.008640
H -3.677672 -1.424527 0.009201
H -1.602312 -2.817818 0.002451
H 3.358709 1.636217 0.370281
H 4.062575 0.100604 0.263051
H -0.265145 2.576697 -0.015336

[M-O]-
E = -564.552058
C -1.391754 1.190580 0.000592
C -0.257703 0.301200 0.001987
C -0.303557 -1.106886 -0.002191
C -1.583942 -1.718793 0.007437
C -2.715202 -0.898915 0.016412
C -2.647206 0.505576 0.013172
N 0.848461 -1.879176 -0.020313
N 2.054653 -1.213813 0.008787
C 2.098791 0.097954 -0.011368
N 1.002263 0.893963 -0.019103
N 3.353797 0.733536 -0.064616
O -1.201274 2.458846 -0.015947
H -3.559799 1.104963 0.015998
H -3.702653 -1.371008 0.024203
H -1.654138 -2.805866 0.006108
H 3.441488 1.489839 0.609909
H 4.077660 0.037020 0.085116
H 0.996866 1.908424 -0.103288

NH2
E = -55.872209
N 0.000000 -0.000000 0.143266
H -0.000000 0.807952 -0.501432
H -0.000000 -0.807952 -0.501432

O
E = -75.077162
O 0.000000 0.000000 0.000000

OH
E = -75.740615
O 0.000000 0.000000 0.108808
H 0.000000 0.000000 -0.870466

M
E = -639.643497
C 0.000000 0.784630 0.000000
C -0.452602 2.121181 0.000000
C -1.812753 2.366265 0.000000
C -2.750676 1.303716 0.000000
C -2.320365 -0.009430 0.000000
C -0.936551 -0.275279 0.000000
H 0.291179 2.913778 0.000000
H -2.170264 3.396105 0.000000
H -3.817620 1.523413 0.000000
H -3.008149 -0.850968 0.000000
N -0.463970 -1.598256 0.000000
N 0.831622 -1.854386 0.000000
O -1.269183 -2.555943 0.000000
C 1.698392 -0.825527 0.000000
N 1.346838 0.491241 0.000000
N 3.019435 -1.078579 0.000000
O 2.273073 1.406685 0.000000
H 3.632577 -0.272797 0.000000
H 3.351008 -2.028942 0.000000

M-
E = -639.701355
C 0.331279 -0.713865 -0.009256
C 1.287705 -1.733891 -0.003522
C 2.655522 -1.406231 -0.000639
C 3.060145 -0.066090 -0.001493
C 2.113034 0.965791 -0.003009
C 0.737664 0.652728 -0.006243

H 0.928680 -2.760104 -0.000377
H 3.400484 -2.203905 0.002651
H 4.123064 0.183646 0.000773
H 2.393284 2.015746 0.001405
N -0.226375 1.646481 0.003958
N -1.569856 1.348526 -0.015785
O 0.103831 2.891052 0.026966
C -1.891714 0.063193 -0.026075
N -1.027244 -0.990296 -0.007723
N -3.219461 -0.299103 -0.081594
O -1.494649 -2.246451 0.037270
H -3.326376 -1.279861 0.174534
H -3.853857 0.378606 0.316550

"blue, 2"
E = -639.715583
C 1.946679 -0.953388 -0.001466
N 0.636921 -1.413083 0.119183
C -0.303344 -0.506080 0.161930
C -0.083341 0.892732 0.066285
N 1.261109 1.284895 0.004110
N 2.246589 0.417737 -0.013716
C -1.726797 -1.020366 0.360265
C -2.775694 -0.011110 -0.005079
C -2.471543 1.323074 -0.121153
C -1.141221 1.817528 -0.040121
O 1.533142 2.522385 -0.040178
N 2.915627 -1.825094 -0.087170
O -1.913677 -2.253478 -0.344742
H 3.789697 -1.298849 -0.166175
H -0.913291 2.875148 -0.132457
H -3.277213 2.038499 -0.303419
H -3.801569 -0.371460 -0.090609
H -1.817514 -1.244276 1.448700
H -1.025984 -2.655842 -0.357504

"blue, 4"
E = -639.687487
C -1.258340 1.780517 0.003007
C -0.146676 0.900848 0.096041
C -0.348546 -0.479243 0.118711
C -1.713811 -1.140122 0.286576
C -2.786210 -0.140657 -0.134015
C -2.550365 1.217404 -0.173405
N 1.188738 1.333550 0.034981
N 2.221405 0.501504 -0.001163
C 2.000206 -0.865974 -0.043368
N 0.675184 -1.312616 0.006098
N 2.947368 -1.748777 -0.121803
O 1.420313 2.573401 0.016436
O -1.745949 -2.405238 -0.122481
H 3.838420 -1.251298 -0.157455
H -1.084248 2.851777 -0.031549
H -3.379269 1.902738 -0.371405
H -3.781671 -0.552901 -0.305996
H -1.789025 -1.047828 1.437430
H 0.394456 -2.300057 -0.070739

"green, 2"
E = -639.680216
N 0.738759 -1.405535 -0.206714
C -0.468105 -0.869851 -0.109822
C -0.721925 0.538878 0.007862
N 0.395056 1.399998 -0.094710
N 1.598719 0.944336 -0.191757
C 1.821273 -0.508893 -0.030166
C -2.016604 1.071619 0.121975
C -3.113856 0.225000 0.124996
C -2.907662 -1.176109 -0.015847
C -1.643547 -1.706175 -0.140484
O 0.196250 2.655430 -0.140450
N 2.893127 -0.878196 -0.926317
H -1.481576 -2.779075 -0.247500
H -3.772032 -1.845153 -0.025061
H -4.122312 0.627663 0.224539
H -2.120637 2.151686 0.200216
H 3.399282 0.002905 -1.104283
O 2.329424 -0.676700 1.341273
H 2.814818 0.141089 1.530903

"black, 4"
E = -639.691269
N -1.034882 -0.903695 0.291584
C 0.315426 -0.706995 0.087059
C 0.736670 0.651366 0.028092
N -0.228077 1.653133 0.074573
N -1.572587 1.399263 -0.076538
C -1.963415 0.112552 -0.035320
C 2.110166 0.945822 -0.068036
C 3.044131 -0.095104 -0.122021
C 2.622982 -1.429609 -0.087976
C 1.253749 -1.738943 0.016888
O 0.137924 2.883040 0.122981
N -3.176573 -0.352999 -0.243976
O -1.527952 -2.205768 0.093623
H 0.906489 -2.768746 0.055567
H 3.353539 -2.238766 -0.136773
H 4.107337 0.141138 -0.195613
H 2.401681 1.992370 -0.101626
H -3.806845 0.413299 -0.465872
H -2.475399 -1.961923 -0.120124

"green, 4"
E = -639.692205
C -1.594555 -1.719614 -0.173060
C -0.455732 -0.861047 -0.127306
C -0.719012 0.534267 -0.003267
C -2.009443 1.042118 0.112105
C -3.101016 0.169309 0.092478
C -2.881357 -1.214540 -0.064409
N 0.407254 1.441801 -0.085326
N 1.598961 1.022352 -0.155821
C 1.848381 -0.444064 0.092799
N 0.785567 -1.371106 -0.235644
O 0.133052 2.686666 -0.125096
N 2.999730 -0.725661 -0.776761
O 2.027060 -0.664542 1.401943
H -1.408319 -2.788530 -0.279119
H -3.733349 -1.897660 -0.091420
H -4.114541 0.560608 0.188092
H -2.131530 2.119099 0.201304
H 3.752679 -0.103770 -0.481921
H 3.289976 -1.677021 -0.552903

"blue, 3"
E = -639.685678
n 0.614122 -1.313062 0.061893
h -0.035893 -2.258564 -0.079751
o -1.517740 -2.409162 -0.091543
c -1.687143 -1.125586 0.345113
c -2.752466 -0.198465 -0.214328
c -2.547191 1.166863 -0.255466
c -1.288509 1.787298 -0.000778
c -0.156316 0.946319 0.168444
c -0.343272 -0.429525 0.218041
n 1.183092 1.354278 0.063122
n 2.186352 0.495377 -0.011262
c 1.935420 -0.881385 -0.065273
n 2.886443 -1.751114 -0.216434
h 3.765706 -1.236646 -0.298284
o 1.438558 2.590410 0.030991
h -1.157146 2.863757 -0.065853
h -3.380397 1.820395 -0.528274
h -3.723383 -0.631105 -0.460943
h -1.888616 -1.099283 1.461788

"green, 3"
E = -639.646048
h 3.300153 -1.095460 0.552746
n 3.070535 -0.779672 -0.656902
o 2.402822 -0.628515 1.280463
c 1.783758 -0.418593 -0.024260

n 1.539289 1.004545 -0.231248
n 0.326218 1.425490 -0.102383
o 0.087396 2.672615 -0.156830
c -0.763603 0.530850 0.028608
c -0.472241 -0.862499 -0.156942
n 0.757003 -1.345730 -0.293238
c -2.067910 1.019316 0.203712
c -3.140535 0.140573 0.193441
c -2.897649 -1.243061 -0.029012
c -1.621447 -1.729738 -0.210198
h -1.434295 -2.791270 -0.375307
h -2.200558 2.091332 0.334182
h -4.157012 0.506528 0.341340
h -3.742578 -1.936242 -0.054566
h 3.538998 0.118793 -0.833149

"black, 3"
E = -639.688563
o -1.655674 -2.139070 0.000001
h -2.755157 -1.576537 -0.000006
n -1.034692 -0.909152 0.000008
c 0.314619 -0.724190 0.000003
c -1.928959 0.152563 0.000000
n -1.556276 1.430331 -0.000002
n -0.189875 1.658983 0.000002
c 0.753675 0.636731 0.000001
c 2.134941 0.911717 -0.000002
c 3.058954 -0.142324 -0.000003
c 2.621700 -1.471712 -0.000002
c 1.246320 -1.768744 0.000000
n -3.163724 -0.352923 -0.000004
o 0.192979 2.884501 0.000002
h -3.932057 0.301646 -0.000019
h 2.442447 1.954054 -0.000003
h 4.127227 0.081890 -0.000005
h 3.346577 -2.287690 -0.000004
h 0.876988 -2.791727 0.000001

"black, 5"
E = -639.670455
n -1.031807 -0.976579 0.405028
o -1.461653 -2.293204 -0.681500
c 0.290234 -0.681681 0.227865
c -1.950180 0.066473 0.180608
n -1.538387 1.382076 0.094881
n -0.245860 1.657164 -0.018977
c 0.720277 0.650419 -0.024821
c 2.085747 0.952947 -0.202297
c 3.026299 -0.061619 -0.131588
c 2.615772 -1.393124 0.127803
c 1.276662 -1.694988 0.312230
h 4.083515 0.165871 -0.276586
h 2.357509 1.988100 -0.395394
n -3.211552 -0.266589 0.099915
h -3.751247 0.583551 -0.067254
o 0.115778 2.879092 -0.112276
h -2.417094 -2.123287 -0.579145
h 0.938109 -2.714797 0.486500
h 3.360586 -2.189617 0.177355

"black, 6"
E = -639.692515
n 0.920617 -0.850961 0.000436
o 4.965911 -1.312323 -0.001040
c -0.415301 -0.804791 0.000155
c 1.568629 0.347883 0.000501
n 0.888484 1.595450 -0.000397
n -0.407317 1.608236 -0.000238
c -1.162126 0.410925 0.000112
c -2.565793 0.446616 0.000166
c -3.275748 -0.743866 0.000085
c -2.568254 -1.975662 -0.000193
c -1.189659 -2.012970 -0.000164
h -4.365931 -0.735207 0.000230
h -3.058712 1.416193 0.000252
n 2.881800 0.405838 0.001312
h 3.166201 1.384892 0.001406
o -1.019912 2.713875 -0.000626
h 4.125123 -0.728934 0.000299
h -0.641269 -2.954807 -0.000241
h -3.128979 -2.913302 -0.000385

"green, 1"
E = -639.670523
c 1.757362 -0.490731 -0.273062
o 2.272880 -0.636397 1.555981
n 1.579791 0.920732 -0.360376
n 0.388407 1.392905 -0.180812
c -0.725757 0.537091 -0.020832
c -0.487918 -0.856562 -0.219353
n 0.727703 -1.371242 -0.427455
n 3.016402 -0.927096 -0.636447
c -2.005328 1.065523 0.215463
c -3.096718 0.212589 0.257028
c -2.903844 -1.177094 0.034019
c -1.649580 -1.697509 -0.206179
h -1.493265 -2.763657 -0.370218
h -3.766877 -1.846763 0.057759
h -4.095373 0.605129 0.451126
h -2.103463 2.140041 0.353247
o 0.202552 2.646532 -0.189594
h 3.614435 -0.092761 -0.609817
h 2.725662 0.209999 1.699930

"green, 5"
E = -639.687529
n 0.815388 -1.337613 -0.262095
c 1.871146 -0.396849 0.096617
n 3.099018 -0.688604 -0.662870
h 3.694827 -1.206387 -0.019612
h 2.850966 -1.333044 -1.411989
o 1.987306 -0.613061 1.414983
n 1.588074 1.064917 -0.172777
n 0.385787 1.457724 -0.100761
c -0.725792 0.532322 0.000976
c -0.439311 -0.853763 -0.136310
o 0.081844 2.694418 -0.166927
c -2.023967 1.015874 0.130140
c -3.098885 0.122008 0.113916
c -1.558871 -1.734046 -0.184542
c -2.855144 -1.254985 -0.058794
h -1.353179 -2.798316 -0.301565
h -3.694016 -1.954225 -0.083109
h -4.118697 0.492935 0.223611
h -2.166026 2.089858 0.225714

"green, 6"
E = -639.689136
c -1.861516 -0.417940 -0.121941
n -3.132239 -0.683894 0.557352
o -1.915437 -0.679823 -1.435100
n -1.605877 1.035494 0.177907
n -0.406848 1.443584 0.092347
c 0.711706 0.532328 -0.020077
c 0.441318 -0.854575 0.157169
n -0.808313 -1.348187 0.278334
c 1.575979 -1.715012 0.238110
c 2.863747 -1.221164 0.093699
c 3.090147 0.153419 -0.127454
c 2.003848 1.030345 -0.167759
o -0.122432 2.681272 0.175299
h 1.387461 -2.778112 0.389918
h 3.712535 -1.907414 0.140437
h 4.104643 0.533314 -0.253619
h 2.132237 2.102916 -0.294693
h -3.037252 -0.444763 1.544927
h -3.275120 -1.690914 0.499371

"green, 7"
E = -639.671948
c 1.912101 -0.406954 0.246694

n 2.205824 -0.781976 -1.459255
o 2.940808 -0.628942 0.923048
n 1.607842 1.067240 0.118944
n 0.406570 1.467652 0.050255
c -0.712845 0.548414 0.027783
c -0.428819 -0.837965 0.172564
n 0.813858 -1.333082 0.328342
c -1.552954 -1.720319 0.183026
c -2.845054 -1.239045 0.046298
c -3.085492 0.142992 -0.104953
c -2.010456 1.035237 -0.108089
o 0.115745 2.707506 0.002048
h -1.348732 -2.784360 0.303767
h -3.685550 -1.936460 0.053889
h -4.104373 0.516619 -0.213018
h -2.147890 2.109467 -0.206982
h 1.347658 -0.878855 -2.013863
h 2.588923 -1.727934 -1.372508

“blue, 1”
E = -639.682794
o -1.447654 2.995914 1.099592
c -1.713919 0.870566 -0.560780
n 0.647546 1.323026 -0.435218
c -0.356343 0.449619 -0.341995
c -0.171901 -0.927499 -0.006938
n 1.171426 -1.354329 0.188017
n 2.165237 -0.537037 0.078770
c 1.914763 0.835396 -0.226166
n 2.951926 1.623384 -0.302982
o 1.382338 -2.567368 0.469963
c -1.232874 -1.822877 0.133617
c -2.537789 -1.371299 -0.060624
c -2.763132 -0.022671 -0.408308
h -1.885975 1.895173 -0.882292
h -0.587480 2.755606 0.693076
h -3.784857 0.326221 -0.570130
h -3.375862 -2.060155 0.047907
h -1.010945 -2.854939 0.395423
h 3.781873 1.057000 -0.113365

“blue, 5”
E = -639.680366
c -1.703423 -1.138485 0.088994
h -1.230472 -0.781794 1.295858
o -1.846502 -2.395120 -0.083774
c -2.808931 -0.149021 0.003349
c -2.561854 1.209091 -0.039843
c -1.257206 1.755681 -0.018357
c -0.149784 0.890397 0.007600
c -0.355163 -0.524295 0.019323
n 0.713707 -1.328512 -0.119773
c 2.029305 -0.845371 -0.004781
n 2.233449 0.509951 0.056373
n 1.164937 1.332383 -0.000177
o 1.387496 2.585506 -0.038040
n 2.979427 -1.733405 0.011557
h 3.869223 -1.234887 0.063864
h 0.540441 -2.326576 -0.203655
h -1.080271 2.826676 -0.041115
h -3.406683 1.900099 -0.094201
h -3.818502 -0.557506 -0.019811

“blue, 6”
E = -639.727602
c -1.762879 -1.108029 0.055292
h -0.294963 -0.678464 1.503932
o -1.912614 -2.339353 -0.036168
c -2.812468 -0.136178 -0.051089
c -2.531126 1.230127 -0.090320
c -1.219339 1.753715 -0.059135
c -0.146748 0.885380 0.077047
c -0.358286 -0.565361 0.391161
n 0.682654 -1.330417 -0.262409
c 1.980444 -0.880131 -0.062664
n 2.212508 0.483270 0.095983
n 1.170351 1.314320 0.026507
o 1.415309 2.560327 -0.056139
n 2.961752 -1.734240 -0.082074
h 3.832438 -1.203930 -0.012168
h 0.573104 -2.336421 -0.208270
h -1.029141 2.816591 -0.181090
h -3.360125 1.933345 -0.202266
h -3.831325 -0.506579 -0.169496

(H2O)2
E = -152.850988
O -1.513832 -0.001866 -0.121826
H -1.920175 0.011833 0.751916
H -0.555426 0.000554 0.044922
O 1.387373 0.001494 0.111079
H 1.743470 -0.771134 -0.345378
H 1.743803 0.761724 -0.365488

(H2O)3
E = -229.285205
O 1.459642 -0.683380 0.085931
H 1.993287 -1.111866 -0.592566
H 0.574861 -1.098715 0.038073
O -1.327983 -0.912034 0.094666
H -1.218441 0.057790 0.016223
H -1.978737 -1.161685 -0.570685
O -0.129540 1.602737 -0.111783
H -0.045514 2.232789 0.612970
H 0.657590 1.023107 -0.054523

H2O
E = -76.423418
O -0.000000 -0.000000 0.117802
H -0.000000 0.764237 -0.471206
H -0.000000 -0.764237 -0.471206

OH-.(H2O)2
E = -228.728586
O -0.002130 0.675229 0.046206
H -0.000393 1.504161 -0.446596
H 1.427313 0.110324 0.077426
O 2.376740 -0.311405 0.067988
H 2.262179 -1.068923 -0.515838
H -1.386460 0.031032 -0.078563
O -2.335884 -0.394720 -0.096699
H -2.612452 -0.329427 0.823614

OH
E = -75.740615
O 0.000000 0.000000 0.108808
H 0.000000 0.000000 -0.870466

OH-.H2O
E = -152.275105
O -1.241966 0.091841 -0.067543
H -1.563683 -0.578468 0.548912
H 0.120771 -0.033772 -0.068033
O 1.236680 -0.097562 -0.053888
H 1.485202 0.658005 0.490568

OH-
E = -75.808426
O 0.000000 0.000000 0.107739
H 0.000000 0.000000 -0.861915

TPZ.H
E = -640.213930
N -1.025171 -0.907258 0.337741
C 0.358462 -0.690077 0.109569
C 0.779305 0.654152 0.034473
N -0.191395 1.666973 0.108780
N -1.524825 1.372516 -0.135956
C -1.889975 0.130576 -0.038537
C 2.141200 0.956430 -0.096578
C 3.071633 -0.081958 -0.158053

C 2.650395 -1.414324 -0.096636
C 1.289804 -1.722577 0.038962
O 0.135463 2.889629 0.173173
N -3.211052 -0.205287 -0.194842
O -1.484358 -2.197112 -0.020724
H 0.955878 -2.756108 0.083771
H 3.378162 -2.223363 -0.151373
H 4.131335 0.151862 -0.257853
H 2.432854 2.002442 -0.146470
H -3.394266 -1.122362 -0.579890
H -3.774650 0.549270 -0.564788
H -1.675999 -2.623810 0.827745

TPZ.H
E = -640.212813
C -1.312210 -1.713210 -0.099096
C -0.365668 -0.691235 -0.118548
C -0.769048 0.658688 -0.018202
C -2.130240 0.972357 0.102496
C -3.074150 -0.055703 0.122800
C -2.671286 -1.391329 0.024721
N 0.206594 1.665593 -0.059320
N 1.555237 1.362578 0.034376
C 1.899933 0.110707 -0.016754
N 1.016613 -0.935012 -0.288263
O 1.468754 -2.225964 0.019555
O -0.114842 2.891303 -0.065282
N 3.206879 -0.241326 0.200949
H -0.989396 -2.747056 -0.196609
H -3.411020 -2.191108 0.035949
H -4.131920 0.189981 0.214257
H -2.410457 2.019913 0.176779
H 1.375347 -2.334209 0.985701
H 3.850593 0.538511 0.173385
H 3.524308 -1.103224 -0.222338

TPZ.H
E = -640.202043
C -1.331002 -1.717780 -0.005953
C -0.376594 -0.705826 -0.005762
C -0.775233 0.652907 -0.011116
C -2.139995 0.986422 -0.007289
C -3.087097 -0.036110 -0.011115
C -2.689312 -1.380601 -0.011860
N 0.189291 1.650852 0.012214
N 1.542542 1.274752 -0.168340
C 1.900095 -0.056042 -0.017672
N 1.003573 -1.026968 0.011639
O 1.407611 -2.282161 0.050257
O -0.030796 2.895804 0.083101
N 3.213419 -0.389930 0.073910
H -0.979949 -2.746648 0.004173
H -3.438624 -2.171414 -0.013828
H -4.146561 0.219016 -0.015728
H -2.419830 2.036747 -0.003349
H 3.316573 -1.404300 0.002066
H 3.880105 0.180139 -0.430609
H 2.126829 1.958555 0.309057

TPZ.H
E = -640.217614
C -1.324637 -1.750708 -0.019850
C -0.360664 -0.738205 0.050745
C -0.745057 0.622298 0.080779
C -2.105066 0.958333 0.016789
C -3.058693 -0.056250 -0.060005
C -2.673139 -1.405608 -0.072257
N 0.268522 1.563836 0.254721
N 1.580947 1.272038 -0.016361
C 1.918010 0.004963 -0.010702
N 1.007757 -1.044860 0.085405
O 1.435590 -2.265509 0.055832
O -0.071807 2.847619 -0.200288
N 3.221041 -0.377876 -0.081633
H -0.986311 -2.783659 -0.038321
H -3.428452 -2.188825 -0.128346

H -4.114661 0.208561 -0.111790
H -2.394905 2.005284 0.022776
H 3.372098 -1.354752 -0.301485
H 3.893872 0.316248 -0.368311
H 0.295686 3.439363 0.473206

TPZ.H.H2O
E = -716.642381
C 0.997700 -1.984063 0.005989
C 0.522224 -0.674230 -0.029438
C 1.425958 0.409266 -0.068245
C 2.808054 0.167246 -0.070776
C 3.276694 -1.145779 -0.033900
C 2.379101 -2.218482 0.006790
N 0.920543 1.714839 -0.117271
N -0.424089 1.971180 0.076092
C -1.239973 0.960111 0.157739
N -0.861878 -0.370446 -0.085643
O -1.709795 -1.360411 0.450599
O 1.692140 2.713096 -0.239919
N -2.546747 1.171987 0.491662
O -4.407926 -0.956492 -0.523036
H 0.292127 -2.811155 0.023846
H 2.747742 -3.243393 0.032847
H 4.350421 -1.332220 -0.037322
H 3.477587 1.022936 -0.100552
H -3.255995 0.538900 0.122334
H -2.795662 2.151999 0.521185
H -3.629121 -1.529321 -0.464275
H -4.805334 -1.134859 -1.383252
H -1.610468 -1.329778 1.421204

TPZ.H.H2O
E = -716.644736
C 2.638618 0.196566 0.024891
C 1.251253 0.285656 0.100191
C 0.460016 -0.881478 0.039306
C 1.068311 -2.138036 -0.080887
C 2.459575 -2.221627 -0.146965
C 3.241305 -1.062458 -0.100231
N -0.933800 -0.744904 0.112082
N -1.536718 0.475723 -0.110471
C -0.792565 1.538540 -0.027914
N 0.563103 1.505455 0.315240
O 1.282711 2.668191 -0.036323
O -1.694334 -1.765096 0.168760
N -1.360953 2.773981 -0.171511
O -4.576626 -1.168843 -0.042384
H 3.241173 1.100706 0.057276
H 4.327104 -1.130446 -0.159184
H 2.935088 -3.197681 -0.239085
H 0.431152 -3.017664 -0.121574
H -0.771592 3.504673 -0.546322
H -2.315805 2.747246 -0.505835
H -3.609200 -1.274088 0.015003
H -4.920271 -2.057938 0.098116
H 1.507841 3.076409 0.813461

TPZ.H.H2O
E = -716.649040
C 2.257700 1.254074 -0.111777
C 1.033840 0.621756 0.088036
C 0.951504 -0.786798 0.084952
C 2.106905 -1.557208 -0.101912
C 3.333049 -0.918332 -0.292394
C 3.409564 0.478207 -0.303768
N -0.301803 -1.383079 0.288553
N -1.461809 -0.645421 0.098867
C -1.370409 0.657446 0.133153
N -0.163662 1.321363 0.382816
O -0.163464 2.684078 0.018819
O -0.428244 -2.638540 0.417630
N -2.487627 1.420743 0.020195
O -4.203918 -0.853260 -0.590306
H 2.310451 2.339759 -0.121907
H 4.367011 0.974255 -0.459741

H 4.232935 -1.515776 -0.436547
H 2.010292 -2.639909 -0.092883
H -2.370658 2.365246 -0.315076
H -3.339159 0.913133 -0.221532
H -3.322845 -1.202109 -0.346358
H -4.845639 -1.420098 -0.148558
H -0.105981 3.157153 0.862696

TPZ.H.(H2O)2
E = -793.077624
C -1.297998 2.001589 -0.045381
C -0.892934 0.669355 -0.114152
C -1.847344 -0.363415 -0.001855
C -3.203772 -0.052967 0.173237
C -3.600118 1.282324 0.236141
C -2.652255 2.306975 0.130813
N -1.421670 -1.699926 -0.069599
N -0.082215 -2.015431 -0.172854
C 0.775316 -1.044011 -0.218096
N 0.460581 0.310993 -0.328304
O 1.371689 1.199607 0.293836
O -2.245568 -2.662018 -0.010256
N 2.132942 -1.354517 -0.215222
O 4.330159 -0.050796 1.296428
O 3.732223 1.147816 -1.082685
H -0.551053 2.788251 -0.114561
H -2.963119 3.349923 0.185566
H -4.654101 1.523988 0.371727
H -3.910747 -0.873908 0.260029
H 2.672581 -0.823588 -0.898617
H 2.260532 -2.355877 -0.324379
H 4.194078 0.896061 -0.251539
H 4.339737 1.682888 -1.604152
H 3.492522 -0.536779 1.166404
H 4.243584 0.389129 2.150464
H 2.055122 1.396093 -0.381920

TPZ.H.(H2O)2
E = -793.073337
C -0.040967 -2.639812 0.015964
C 0.016878 -1.247474 0.027822
C 1.265941 -0.588707 0.051539
C 2.452941 -1.336228 0.065114
C 2.386900 -2.729315 0.052472
C 1.148629 -3.380390 0.025431
N 1.287436 0.809563 0.070916
N 0.146627 1.555627 -0.113219
C -0.996902 0.932383 -0.177907
N -1.143341 -0.437682 0.070788
O -2.321143 -1.037984 -0.405121
O 2.385962 1.448557 0.166402
N -2.122193 1.626807 -0.499193
O -4.669448 0.430533 0.511174
O 2.118610 4.383879 0.060364
H -1.007509 -3.137575 0.008915
H 1.100365 -4.468759 0.017852
H 3.308804 -3.310099 0.064022
H 3.398081 -0.800071 0.082602
H -3.023815 1.307266 -0.145138
H -1.979258 2.627571 -0.543269
H -4.223624 -0.427201 0.552317
H -5.163563 0.514628 1.335001
H 2.107135 3.409193 0.080806
H 3.044898 4.612229 0.195029
H -2.253572 -1.070013 -1.378338

TPZ.H.(H2O)2
E = -793.086377
N -0.520735 1.498026 0.341417
C -1.526726 0.537603 0.081599
C -1.122404 -0.817258 0.069733
N 0.237737 -1.100363 0.223097
N 1.190107 -0.123611 0.083027
C 0.808220 1.125309 0.113630
C -2.073846 -1.834513 -0.091019
C -3.418712 -1.496437 -0.244940
C -3.815532 -0.154857 -0.247088
C -2.869536 0.866763 -0.082642
O 0.669889 -2.304217 0.288584
N 1.739128 2.099556 0.017607
O -0.835778 2.821783 -0.024512
O 4.230258 0.692462 -0.308128
O 3.556319 -2.036461 -0.096021
H -3.172580 1.910701 -0.089238
H -4.865429 0.106345 -0.375885
H -4.160100 -2.285366 -0.368844
H -1.729554 -2.865478 -0.095024
H 1.441694 3.020517 -0.263927
H 2.693415 1.774611 -0.181997
H 2.587411 -2.111621 0.039229
H 3.949182 -2.614263 0.567428
H 3.961835 -0.248957 -0.199522
H 4.919106 0.678549 -0.981029
H -1.002935 3.271517 0.817738

[TPZ-OH]
E = -563.816163
C 2.231943 -0.282006 0.000000
C 0.819934 -0.467937 0.000000
C 0.000000 0.693794 0.000000
C 0.534082 1.984082 0.000000
C 1.917906 2.128402 0.000000
C 2.762988 0.993594 0.000000
N -1.413584 0.496436 0.000000
N -1.923267 -0.704392 0.000000
C -1.046648 -1.791374 0.000000
N 0.304442 -1.714985 0.000000
O -2.158923 1.493009 0.000000
N -1.590095 -3.008960 0.000000
H 2.858960 -1.172182 0.000000
H 3.844317 1.130860 0.000000
H 2.354070 3.126958 0.000000
H -0.138631 2.837970 0.000000
H -2.611035 -2.895713 0.000000

[TPZ-OH]
E = -563.814403
C -1.315651 1.456983 0.000012
C -0.317077 0.469717 0.000033
C -0.646794 -0.903469 0.000141
C -1.998467 -1.285944 -0.000057
C -2.992237 -0.313552 -0.000204
C -2.646137 1.052740 -0.000115
N 0.322885 -1.910859 0.000297
N 1.557655 -1.618209 0.000430
C 2.004156 -0.274450 -0.000648
N 1.039001 0.789952 -0.000042
O 1.435031 1.994848 0.000517
N 3.266711 -0.045985 -0.000490
H -1.030039 2.505847 0.000010
H -3.432761 1.807274 -0.000210
H -4.041631 -0.606368 -0.000332
H -2.230632 -2.349995 -0.000048
H 3.424290 0.968012 0.000113

TPZ.H2O
E = -716.076449
N 0.867992 -0.395596 -0.039488
C -0.484342 -0.672015 -0.017832
C -1.411873 0.392148 0.006749
N -0.920127 1.710668 0.009230
N 0.373200 1.948844 -0.010276
C 1.245039 0.912296 -0.035753
C -2.797358 0.144485 0.028915
C -3.243144 -1.164150 0.026236
C -2.315824 -2.233839 0.001599
C -0.952356 -2.002890 -0.020651
O -1.713485 2.675053 0.030871
N 2.550776 1.217454 -0.066786
O 1.748781 -1.366950 -0.068907
H -0.219456 -2.804544 -0.039747
H -2.682513 -3.260435 0.000171

H -4.312076 -1.372680 0.043439
H -3.474735 0.994168 0.047658
H 3.283012 0.503073 -0.028499
H 2.790621 2.195875 -0.022529
H 3.538232 -1.341599 0.136777
O 4.440735 -0.959213 0.192091
H 4.924931 -1.330772 -0.554054

TPZ.H2O
E = -716.072423
N 0.580968 1.572699 0.000013
C 1.240872 0.362142 -0.000037
C 0.486964 -0.836058 0.000116
N -0.908846 -0.748081 0.000356
N -1.519517 0.417176 0.000286
C -0.783694 1.541437 0.000087
C 1.131954 -2.090145 0.000073
C 2.512322 -2.131832 -0.000132
C 3.268306 -0.931947 -0.000276
C 2.650960 0.303938 -0.000226
O -1.612290 -1.794536 0.000619
N -1.403916 2.732967 -0.000193
O 1.197089 2.715686 -0.000020
H 3.202845 1.240266 -0.000335
H 4.357014 -0.985508 -0.000434
H 3.024328 -3.093241 -0.000181
H 0.519597 -2.987834 0.000202
H -0.812282 3.554162 0.000483
H -2.410466 2.774844 0.000885
H -3.581278 -1.344010 -0.000311
O -4.542743 -1.196788 -0.000724
H -4.923135 -2.082116 -0.000177

TPZ.H2O
E = -716.076335
N -0.146446 1.392734 0.005968
C 1.015439 0.654196 -0.000006
C 0.940126 -0.759466 0.003344
N -0.321145 -1.367647 0.011702
N -1.422453 -0.638226 0.013781
C -1.329765 0.708250 0.012440
C 2.113244 -1.541870 -0.001836
C 3.340487 -0.908815 -0.010442
C 3.417607 0.507398 -0.013773
C 2.277618 1.287373 -0.008604
O -0.429276 -2.616536 0.017398
N -2.446926 1.445426 0.019185
O -0.159403 2.693066 0.005264
H 2.304603 2.373894 -0.010979
H 4.395030 0.989961 -0.020649
H 4.254901 -1.500656 -0.014867
H 2.012554 -2.623966 0.000811
H -2.314202 2.448792 0.010400
H -3.352874 0.985242 0.021551
O -4.309407 -0.774576 -0.085311
H -3.446945 -1.220967 -0.021292
H -4.958129 -1.376346 0.295029

TPZ-.H2O
E = -716.141963
N 0.901147 -0.303092 -0.236526
C -0.435690 -0.658133 -0.114840
C -1.394640 0.381145 0.046431
N -0.961528 1.697677 0.078129
N 0.371151 2.021498 -0.033290
C 1.224390 1.019902 -0.202118
C -2.759712 0.055600 0.170894
C -3.158686 -1.286016 0.134550
C -2.212861 -2.304925 -0.024369
C -0.847397 -1.993149 -0.150001
O -1.794549 2.664757 0.225605
N 2.549625 1.337303 -0.385552
O 1.838454 -1.257177 -0.425329
H -0.083088 -2.755336 -0.276143
H -2.530400 -3.348600 -0.051692
H -4.217642 -1.532715 0.231571
H -3.466305 0.872150 0.293025
H 3.217742 0.613897 -0.132365
H 2.770457 2.275314 -0.081509
H 3.244731 -1.246760 0.401569
O 4.184227 -1.138800 0.750002
H 4.724261 -1.588431 0.090705

TPZ-.H2O
E = -716.142340
N 0.443193 1.582261 -0.008510
C 1.196452 0.415946 -0.011626
C 0.504562 -0.829586 0.001128
N -0.875949 -0.828857 0.019066
N -1.604067 0.332981 0.000066
C -0.913764 1.466010 -0.015719
C 1.241401 -2.032174 0.004580
C 2.639757 -1.986206 -0.004026
C 3.315061 -0.759110 -0.013524
C 2.593769 0.446435 -0.016351
O -1.536448 -1.947385 0.048909
N -1.583982 2.665311 -0.067485
O 1.013693 2.790499 0.025506
H 3.076241 1.420635 -0.020949
H 4.405922 -0.734099 -0.018400
H 3.204849 -2.920065 -0.001759
H 0.688912 -2.967615 0.016294
H -0.957280 3.432502 0.170772
H -2.510138 2.652353 0.334845
H -3.330866 -1.736307 0.010689
O -4.286672 -1.493436 -0.030370
H -4.220018 -0.534593 -0.112580

TPZ-.H2O
E = -716.140523
N 0.114366 1.421363 -0.024130
C -1.032594 0.639564 0.001172
C -0.885704 -0.778360 -0.022648
N 0.383136 -1.326819 -0.061761
N 1.504580 -0.529083 -0.080029
C 1.317553 0.790483 -0.076892
C -2.034886 -1.595458 -0.001484
C -3.303571 -1.006350 0.045416
C -3.442720 0.386940 0.072283
C -2.306112 1.213514 0.050354
O 0.568436 -2.600815 -0.084583
N 2.399430 1.618286 -0.149829
O 0.060242 2.758572 0.018373
H -2.367351 2.298837 0.069481
H -4.435766 0.837865 0.110093
H -4.189185 -1.644428 0.062166
H -1.891672 -2.672575 -0.020244
H 2.162984 2.579123 0.078899
H 3.299184 1.216654 0.080225
O 4.235965 -0.948870 0.278865
H 3.251022 -1.010160 0.181741
H 4.571268 -1.464637 -0.462562

[TPZ-OH].H2O
E = -640.245597
C 1.443329 0.623302 0.000260
N 0.968793 -0.646540 0.000267
C -0.374504 -0.766616 0.000082
C -1.237636 0.364206 -0.000105
N -0.626260 1.656123 -0.000135
N 0.671405 1.784384 0.000050
O -1.354520 2.663011 -0.000247
N 2.764156 0.775978 0.000363
C -0.973417 -2.058769 0.000119
C -2.349757 -2.183877 -0.000043
C -3.178811 -1.038018 -0.000233
C -2.627081 0.240703 -0.000260
H 2.981888 1.778013 0.000274
H -3.241693 1.137292 -0.000405
H -4.262124 -1.154924 -0.000351
H -2.804194 -3.174509 -0.000024
H -0.312397 -2.923911 0.000270

H 4.471940 -2.145539 0.000159
O 4.898029 -1.281344 -0.000099
H 4.159106 -0.644958 0.000112

[TPZ-OH].H2O
E = -640.243954
C -2.721527 0.678500 -0.000035
C -1.298297 0.724897 -0.000040
C -0.595882 -0.512379 0.000003
C -1.252652 -1.745054 0.000039
C -2.643904 -1.751680 0.000041
C -3.374052 -0.539441 0.000000
N 0.825904 -0.448862 0.000002
N 1.453015 0.690939 0.000040
C 0.689228 1.862167 -0.000005
N -0.662983 1.914566 -0.000051
O 1.472140 -1.519261 -0.000035
N 1.348264 3.019277 0.000048
O 4.450681 -1.588099 -0.000032
H -3.258682 1.625481 -0.000065
H -4.463533 -0.570731 -0.000002
H -3.176003 -2.702493 0.000080
H -0.668627 -2.661687 0.000062
H 2.354031 2.811642 0.000109
H 3.484018 -1.494584 -0.000101
H 4.779340 -0.682238 0.000144

[TPZ-OH].H2O
E = -640.245702
C -2.331778 -1.410052 -0.000031
C -1.010095 -0.880564 -0.000100
C -0.866220 0.534242 -0.000025
C -1.964475 1.398235 0.000189
C -3.240439 0.845469 0.000300
C -3.419820 -0.558271 0.000170
N 0.459859 1.051896 -0.000240
N 1.489927 0.252126 -0.000248
C 1.268449 -1.129577 -0.000130
N 0.049766 -1.716277 -0.000182
O 0.631104 2.285942 -0.000386
N 2.335772 -1.926524 0.000016
O 4.387833 0.500682 0.000641
H -2.442335 -2.493192 -0.000113
H -4.429492 -0.968948 0.000241
H -4.110531 1.501359 0.000483
H -1.797087 2.472256 0.000245
H 3.189208 -1.351375 0.000035
H 3.488428 0.868612 -0.000016
H 4.989310 1.252864 -0.000573

[TPZ-OH].H2O
E = -640.243637
C -2.492611 -0.040997 0.011303
C -1.128797 -0.343071 0.004445
C -0.131095 0.666490 -0.012188
C -0.556654 2.024076 -0.023170
C -1.905310 2.325067 -0.017428
C -2.875885 1.295930 0.000212
N -0.687610 -1.701400 0.016173
N 0.581032 -2.006020 -0.001203
C 1.498259 -0.962833 -0.023575
N 1.185384 0.359197 -0.019563
N 2.771176 -1.365155 -0.051094
O -1.543230 -2.603284 0.041413
O 3.745273 1.754904 -0.039289
H 3.371640 -0.529094 -0.069992
H -3.215196 -0.852847 0.024810
H -3.934896 1.552141 0.004411
H -2.227872 3.366120 -0.028116
H 0.205016 2.802115 -0.041865
H 2.801805 1.517031 0.033490
H 3.985839 2.137251 0.812485

[TPZ-OH]-.H2O
E = -640.369575
C -1.569039 0.656532 0.000047
N -1.065811 -0.617156 0.000032
C 0.265014 -0.732599 0.000010
C 1.153039 0.385054 0.000001
N 0.547727 1.666456 0.000003
N -0.738515 1.812041 0.000016
O 1.291671 2.688913 -0.000012
N -2.862909 0.859953 -0.000004
C 0.892141 -2.023973 0.000007
C 2.265565 -2.151259 -0.000005
C 3.115280 -1.013438 -0.000016
C 2.550210 0.252911 -0.000013
H -3.038402 1.863938 -0.000030
H 3.154302 1.157416 -0.000021
H 4.198730 -1.134259 -0.000026
H 2.709777 -3.149475 -0.000009
H 0.238441 -2.896167 0.000012
H -3.340966 -2.064099 0.000178
O -4.195643 -1.613338 -0.000057
H -3.886805 -0.670384 -0.000058

[TPZ-OH]-.H2O
E = -640.363406
C -2.612524 0.912801 -0.000008
C -1.176311 0.828626 0.000048
C -0.639370 -0.497748 0.000086
C -1.440413 -1.651769 0.000083
C -2.819952 -1.522130 0.000032
C -3.395002 -0.221232 -0.000016
N 0.766948 -0.589915 0.000124
N 1.526161 0.453293 0.000137
C 0.933620 1.757844 0.000128
N -0.429518 1.930452 0.000066
O 1.298660 -1.751051 0.000159
N 1.743555 2.780022 0.000220
O 4.142451 -1.565238 -0.000821
H -3.050431 1.910933 -0.000042
H -4.483025 -0.119845 -0.000057
H -3.457333 -2.406446 0.000028
H -0.950849 -2.622881 0.000118
H 2.700005 2.419999 0.000275
H 3.171701 -1.705262 -0.000341
H 4.190746 -0.601513 -0.000625

[TPZ-OH]-.H2O
E = -640.360325
C -2.369628 -1.326829 0.196075
C -1.005263 -0.896931 0.041736
C -0.817588 0.516486 -0.075624
C -1.878070 1.436945 -0.056388
C -3.177530 0.976697 0.084997
C -3.407680 -0.420830 0.213650
N 0.518641 0.958904 -0.189800
N 1.509388 0.121726 -0.217719
C 1.256773 -1.287235 -0.162471
N -0.012165 -1.783173 0.010988
O 0.752869 2.199108 -0.255473
N 2.282151 -2.086617 -0.272002
O 4.261920 0.932489 0.485873
H -2.543512 -2.398581 0.292445
H -4.431991 -0.783837 0.328471
H -4.012933 1.676887 0.100160
H -1.645406 2.495332 -0.151331
H 3.117694 -1.516882 -0.417934
H 3.337943 0.840461 0.168924
H 4.367701 0.168147 1.063942

[TPZ-OH]-.H2O
E = -640.364982
C -2.427530 0.042471 0.031793
C -1.078599 -0.334915 0.003899
C -0.012169 0.605002 -0.056051
C -0.392777 1.986293 -0.080563
C -1.723732 2.355722 -0.052907
C -2.760295 1.390553 0.002154
N -0.706744 -1.710498 0.043871
N 0.523360 -2.094436 0.012621

C 1.553871 -1.116623 -0.069978
N 1.276727 0.236302 -0.087165
N 2.754610 -1.619995 -0.120642
O -1.630006 -2.571871 0.114640
O 3.266388 2.190516 0.097097
H 3.411155 -0.838586 -0.189912
H -3.180944 -0.740534 0.077184
H -3.804969 1.701614 0.021809
H -1.982205 3.417101 -0.074479
H 0.402655 2.730192 -0.121841
H 2.571216 1.481999 -0.005957
H 3.603749 2.048425 0.988423

TPZ.(H2O)2
E = -792.510356
N 0.503034 0.149145 -0.307202
C -0.790633 0.607550 -0.182884
C -1.830425 -0.314740 0.067353
N -1.503652 -1.680585 0.177258
N -0.260956 -2.092726 0.053535
C 0.720505 -1.188440 -0.187095
C -3.163690 0.115248 0.200936
C -3.447087 1.463326 0.081580
C -2.408078 2.390592 -0.173087
C -1.094017 1.979765 -0.305694
O -2.400310 -2.520505 0.396988
N 1.966181 -1.655541 -0.322756
O 1.501066 0.961835 -0.575540
H -0.279942 2.670659 -0.505802
H -2.647311 3.449924 -0.267748
H -4.473820 1.812569 0.182125
H -3.931343 -0.629520 0.393644
H 2.787753 -1.046085 -0.391622
H 2.095032 -2.647199 -0.190565
H 4.381114 0.458741 0.236118
O 4.579375 -0.391713 -0.217282
H 5.267542 -0.811013 0.310852
O 3.512762 1.833348 0.995620
H 2.680577 1.590155 0.520857
H 3.725555 2.726181 0.701350

TPZ.(H2O)2
E = -792.505130
N 1.154962 -0.482234 -0.027582
C -0.003974 -1.231922 -0.012643
C -1.257293 -0.579102 0.004492
N -1.273354 0.821757 0.005870
N -0.161700 1.515235 -0.007113
C 1.030754 0.875756 -0.024061
C -2.458085 -1.315653 0.020093
C -2.394354 -2.695778 0.018320
C -1.139664 -3.353803 0.001109
C 0.045458 -2.642173 -0.014618
O -2.368859 1.441007 0.019982
N 2.130977 1.638806 -0.044279
O 2.326864 -1.062102 -0.047935
H 1.020010 -3.121950 -0.027733
H -1.106991 -4.443359 0.000365
H -3.313021 -3.280724 0.030524
H -3.399533 -0.773163 0.033170
H 3.075588 1.244052 -0.017549
H 1.992447 2.637904 -0.011672
H 4.013223 -0.382620 0.109089
O 4.694226 0.320260 0.158857
H 5.276426 0.172276 -0.595112
H -2.052767 3.451656 0.005823
O -1.967677 4.420014 -0.004515
H -2.875061 4.743600 0.017549

TPZ.(H2O)2
E = -792.513026
C -2.878750 0.932725 0.007340
C -1.508646 0.588324 0.000219
C -1.130489 -0.778352 -0.004082
C -2.107643 -1.796156 -0.001680
C -3.441768 -1.442046 0.004851
C -3.822766 -0.074924 0.009500
N 0.228590 -1.086031 -0.010509
N 1.141357 -0.145304 -0.015223
C 0.771701 1.148846 -0.009182
N -0.534831 1.562367 -0.001537
O -0.802707 2.831341 0.004643
O 0.623864 -2.291848 -0.012534
N 1.721434 2.086570 -0.010862
O 3.578445 -2.019643 -0.058996
H -3.140780 1.987540 0.010853
H -4.881403 0.184882 0.014799
H -4.207621 -2.216614 0.006309
H -1.776656 -2.831265 -0.005873
H 1.406497 3.047277 0.009456
H 2.702731 1.788076 -0.001540
H 2.606554 -2.125295 -0.040253
H 3.924581 -2.710623 0.516378
H 4.007487 -0.234057 0.062692
O 4.243506 0.719971 0.082302
H 5.028051 0.797799 -0.471018

TPZ-.(H2O)2
E = -792.580261
N -0.330265 0.436537 -0.663837
C 0.634976 -0.523493 -0.403689
C 1.854671 -0.092428 0.190793
N 2.027325 1.255479 0.478366
N 1.065539 2.193847 0.166037
C -0.047248 1.740244 -0.385448
C 2.856980 -1.036871 0.482443
C 2.645275 -2.388437 0.183233
C 1.446725 -2.807609 -0.403543
C 0.436544 -1.874828 -0.698991
O 3.103908 1.678180 1.032135
N -1.020258 2.635059 -0.755095
O -1.530972 0.101763 -1.199539
H -0.502864 -2.163668 -1.163782
H 1.289195 -3.861610 -0.637061
H 3.425881 -3.116021 0.411968
H 3.774997 -0.677665 0.939734
H -1.911963 2.183826 -0.934740
H -1.009157 3.507877 -0.248213
H -4.935325 -0.717287 0.671318
O -5.911823 -0.662375 0.797744
H -6.018092 0.074017 1.409410
O -3.160597 -0.744215 0.653355
H -2.531886 -0.414953 -0.074792
H -2.748849 -1.547261 0.989519

TPZ-.(H2O)2
E = -792.581804
N 1.199137 -0.190169 -0.222857
C 0.211945 -1.161386 -0.104434
C -1.138105 -0.735001 0.032008
N -1.409484 0.620699 0.042871
N -0.419475 1.562557 -0.064392
C 0.825588 1.118650 -0.206903
C -2.163985 -1.692930 0.152568
C -1.842650 -3.054110 0.136396
C -0.512578 -3.470364 0.001731
C 0.518618 -2.524482 -0.119423
O -2.626562 1.046450 0.168163
N 1.816972 2.050102 -0.380135
O 2.484622 -0.556609 -0.388701
H 1.562216 -2.807619 -0.226947
H -0.269899 -4.533983 -0.009877
H -2.639985 -3.793348 0.229879
H -3.184957 -1.335890 0.255361
H 2.759332 1.760352 -0.130029
H 1.543370 2.981087 -0.100122
H 3.719557 0.188739 0.399238
O 4.466715 0.780689 0.716908
H 5.156625 0.655835 0.055893
H -2.800562 2.851141 0.113980
O -2.787652 3.835772 0.065443
H -1.845738 3.998693 -0.061944

TPZ-.(H2O)2
E = -792.581278
C -2.973347 0.397391 0.157513
C -1.581471 0.406114 0.030224
C -0.866183 -0.817964 -0.116532
C -1.574402 -2.037645 -0.135977
C -2.966944 -2.029646 -0.010798
C -3.665427 -0.823641 0.135591
N 0.507316 -0.778693 -0.224991
N 1.197586 0.400591 -0.238120
C 0.493180 1.519754 -0.095894
N -0.858078 1.592046 0.055173
O -1.448248 2.774418 0.230458
O 1.197710 -1.880118 -0.334088
N 1.136218 2.724457 -0.122385
O 3.785264 -2.146676 0.461198
H -3.474165 1.355416 0.272646
H -4.751976 -0.828649 0.234052
H -3.510073 -2.976149 -0.027274
H -1.004692 -2.955961 -0.247892
H 0.540807 3.478938 0.207424
H 2.122673 2.716576 0.097032
H 2.857740 -1.997790 0.134056
H 3.667893 -2.295445 1.405821
H 4.353703 -0.178371 0.114839
O 4.089686 0.730142 -0.109480
H 3.129064 0.604323 -0.247909

[TPZ-OH].(H2O)2
E = -716.677961
C 0.936086 -1.228736 0.017566
N 0.794557 0.119626 0.027502
C -0.468351 0.586992 0.015803
C -1.590878 -0.287077 -0.001414
N -1.328528 -1.693032 -0.006174
N -0.106503 -2.151462 0.001588
N 2.176167 -1.705922 0.023272
C -2.900290 0.189885 -0.013127
C -3.101143 1.569366 -0.008367
C -2.005756 2.463912 0.007736
C -0.706568 1.992123 0.019754
O -2.288923 -2.479772 -0.018438
H 2.133991 -2.730232 0.008794
H -3.726519 -0.516297 -0.025880
H -4.117755 1.961608 -0.017707
H -2.191100 3.537725 0.009973
H 0.155631 2.658570 0.029937
O 2.779318 2.338469 -0.009239
O 4.641544 -0.011754 -0.106881
H 3.861522 -0.595668 -0.038430
H 5.226641 -0.288470 0.607269
H 3.662651 1.942457 -0.062970
H 2.210979 1.551494 0.014449

[TPZ-OH].(H2O)2
E = -716.672872
N -1.091639 0.684874 -0.003523
C -0.956463 -0.733514 -0.001355
C 0.366700 -1.258672 -0.000239
C 0.518470 -2.674831 0.001630
C -0.597461 -3.489669 0.002501
C -1.898546 -2.933857 0.001539
C -2.085152 -1.554093 -0.000367
O -2.240033 1.174367 -0.003320
O -2.779629 4.124013 0.007024
H -2.523452 3.187816 0.003271
H -1.944141 4.604452 -0.000428
N -0.050390 1.464838 -0.003752
C 1.212276 0.869162 -0.002749
N 2.265408 1.678989 -0.002370
N 1.454922 -0.463909 -0.000935
O 5.106140 0.726483 0.003397
H 5.033870 -0.234274 0.003027
H 4.183001 1.036047 0.001170
H 1.940282 2.651316 -0.002957
H 1.530252 -3.076696 0.002418
H -0.477931 -4.572913 0.004010
H -2.766661 -3.592235 0.002246
H -3.074099 -1.103126 -0.001248

[TPZ-OH].(H2O)2
E = -716.676602
N -0.593963 1.965722 0.208808
C -1.280371 0.812692 0.091290
C -0.698363 -0.366495 -0.451361
N 0.652414 -0.280155 -0.873976
N 1.327034 0.821626 -0.774070
C 0.688912 1.935190 -0.218752
C -1.408533 -1.563079 -0.568399
C -2.731451 -1.590547 -0.141031
C -3.342700 -0.434496 0.400855
C -2.637503 0.747210 0.518028
O 1.200573 -1.305008 -1.356142
N 1.400634 3.055167 -0.116899
O 1.377289 -0.717065 2.061342
O 3.391374 -1.772139 0.379864
H -3.081242 1.650375 0.933459
H -4.380725 -0.482008 0.729967
H -3.302701 -2.514889 -0.222305
H -0.914745 -2.438637 -0.980778
H 2.341930 2.863694 -0.479648
H 2.791972 -1.669330 -0.384701
H 4.136803 -1.190827 0.189137
H 2.167535 -1.116614 1.645021
H 1.444510 -0.927426 2.998525

[TPZ-OH].(H2O)2
E = -716.675876
C -0.665297 1.985118 0.069978
C -0.450629 0.578775 0.019634
C -1.590395 -0.266301 0.006233
C -2.892816 0.238393 0.038988
C -3.069231 1.616635 0.088575
C -1.953093 2.485461 0.104657
N -1.361712 -1.674018 -0.046411
N -0.154424 -2.167542 -0.051813
C 0.916965 -1.280533 -0.023043
N 0.806429 0.074469 -0.009937
N 2.103758 -1.887438 -0.003623
O -2.344983 -2.435134 -0.082154
O 2.868118 2.072235 -0.262509
O 4.574965 -0.079683 0.201743
H 2.866272 -1.192182 0.035574
H -3.729529 -0.455193 0.025121
H -4.077010 2.029929 0.116186
H -2.113134 3.562754 0.147225
H 0.204133 2.640744 0.089481
H 5.191695 -0.080136 0.941829
H 4.197397 0.820884 0.157082
H 2.963025 2.280363 -1.199987
H 2.180976 1.369913 -0.216792

[TPZ-OH]-.(H2O)2
E = -716.810898
C 1.063227 -1.030148 -0.030347
N 0.757797 0.299817 -0.011988
C -0.542291 0.622800 0.005557
C -1.584764 -0.347101 0.005905
N -1.179958 -1.704930 -0.017790
N 0.070684 -2.044052 -0.036289
N 2.312155 -1.433280 -0.043653
C -2.946273 -0.003887 0.026545
C -3.307333 1.333885 0.046615
C -2.294220 2.328689 0.044504
C -0.955989 1.994418 0.024332
O -2.067678 -2.602435 -0.020712
H 2.327761 -2.451873 -0.064034
H -3.682428 -0.804345 0.025586
H -4.359190 1.619850 0.062715
H -2.580864 3.382484 0.058374
H -0.176332 2.755688 0.020198

O 2.874826 2.318518 -0.163944
O 4.665961 -0.041520 0.082751
H 3.795696 -0.543386 0.005466
H 4.797534 0.031063 1.034848
H 3.641890 1.720440 -0.187885
H 2.132179 1.678761 -0.110664

[TPZ-OH]-.(H2O)2
E = -716.806465
N -0.230628 -1.284907 -0.000301
C 1.010751 -0.618051 -0.000112
C 0.953783 0.809357 0.000056
C 2.222370 1.482100 0.000323
C 3.404268 0.773465 0.000376
C 3.418189 -0.648064 0.000181
C 2.217806 -1.338222 -0.000052
O -0.234954 -2.558480 -0.000289
O -2.886515 -3.629707 0.000393
H -1.958195 -3.316310 0.000113
H -3.372239 -2.796335 0.000063
N -1.355780 -0.649170 -0.000341
C -1.348594 0.774950 -0.000308
N -2.509941 1.376223 -0.000517
N -0.187223 1.501061 -0.000023
O -2.150436 4.171593 0.000517
H -1.191590 4.055714 -0.000136
H -2.439536 3.223703 0.000080
H -3.250556 0.676416 -0.000673
H 2.209415 2.571734 0.000479
H 4.352515 1.315705 0.000576
H 4.364501 -1.188839 0.000217
H 2.174488 -2.424696 -0.000195

[TPZ-OH]-.(H2O)2
E = -716.797743
N -0.999050 1.945930 0.120482
C -1.651653 0.791125 0.046458
C -1.007582 -0.484566 -0.046746
N 0.396953 -0.459825 -0.069772
N 1.070020 0.640290 -0.013515
C 0.375584 1.892308 0.104443
C -1.713472 -1.699181 -0.117368
C -3.097277 -1.681676 -0.099067
C -3.777253 -0.433858 -0.010024
C -3.090956 0.757259 0.059781
O 1.016937 -1.576247 -0.153607
N 1.091663 2.973947 0.193949
O 4.114304 0.562566 -0.535804
O 3.649070 -2.195985 0.434024
H -3.607995 1.714174 0.128407
H -4.869705 -0.423090 0.003089
H -3.661149 -2.612848 -0.153937
H -1.148496 -2.625625 -0.186868
H 2.080450 2.719514 0.162580
H 2.726806 -1.933870 0.205221
H 3.711552 -2.012357 1.378272
H 4.230283 -0.377405 -0.318788
H 3.154320 0.677978 -0.417745

[TPZ-OH]-.(H2O)2
E = -716.800708
C -0.981597 1.983016 -0.197165
C -0.533063 0.624889 -0.221988
C -1.517474 -0.364475 0.044074
C -2.859554 -0.054519 0.299856
C -3.263625 1.274054 0.302311
C -2.305822 2.287771 0.055181
N -1.066468 -1.717413 0.064591
N 0.157436 -2.041310 -0.182191
C 1.089468 -1.020602 -0.505383
N 0.748595 0.315387 -0.480819
N 2.278211 -1.467293 -0.810356
O -1.908577 -2.617195 0.338869
O 2.805685 2.098346 -0.155204
O 4.386741 0.033602 1.213885
H 2.853391 -0.662171 -1.067386
H -3.549959 -0.871785 0.494712
H -4.304115 1.533292 0.498093
H -2.619050 3.334180 0.066049
H -0.245409 2.765611 -0.379482
H 3.863805 -0.721461 0.902823
H 3.907976 0.800588 0.845680
H 3.200248 2.216145 -1.026750
H 2.027896 1.481176 -0.314040

References

[1] R. Meißner, J. Kočišek, L. Feketeová, J. Fedor, M. Fárník, P. Limão-Vieira, E. Illenberger, S. Denifl, *Nat. Commun.* **2019**, *10*, 2388.

[2] Y. Zhao, D. G. Truhlar, *Theor. Chem. Acc.* **2008**, *120*, 215–241.

[3] C. E. Moore, *Moore, CE 1993, Tables Spectra Hydrog. Carbon, Nitrogen, Oxyg. Atoms Ions, CRC Ser. Eval. Data At. Phys. (Boca Raton, FL CRC Press. ISBN 0849374200.* **1993**.

[4] M. J. Frisch, G. W. Trucks, H. B. Schlegel, G. E. Scuseria, M. A. Robb, J. R. Cheeseman, G. Scalmani, V. Barone, G. A. Petersson, H. Nakatsuji, et al., **2016**.

Author Contributions

EAB: Experimental investigation (lead), data curation and analysis (lead), preparation of manuscript (lead).
JA: Experimental investigation (Supporting), data curation and analysis (Supporting), preparation of manuscript (supporting).
ZP: Experimental investigation (Supporting), data curation and analysis (supporting).
FFS: Data interpretation (supporting), preparation of manuscript (supporting).
MO: Computations (lead), data analysis (equal), preparation of manuscript (lead).
SD: Conceived the idea, interpretation of data (lead), data validation (lead), funding acquisition (lead), preparation of manuscript (lead).
